# Imbalanced cellular metabolism compromises cartilage homeostasis and joint function in a mouse model of mucolipidosis type III gamma

**DOI:** 10.1242/dmm.046425

**Published:** 2020-11-18

**Authors:** Lena Marie Westermann, Lutz Fleischhauer, Jonas Vogel, Zsuzsa Jenei-Lanzl, Nataniel Floriano Ludwig, Lynn Schau, Fabio Morellini, Anke Baranowsky, Timur A. Yorgan, Giorgia Di Lorenzo, Michaela Schweizer, Bruna de Souza Pinheiro, Nicole Ruas Guarany, Fernanda Sperb-Ludwig, Fernanda Visioli, Thiago Oliveira Silva, Jamie Soul, Gretl Hendrickx, J. Simon Wiegert, Ida V. D. Schwartz, Hauke Clausen-Schaumann, Frank Zaucke, Thorsten Schinke, Sandra Pohl, Tatyana Danyukova

**Affiliations:** 1 Department of Osteology and Biomechanics, University Medical Center Hamburg-Eppendorf, 20246 Hamburg, Germany; 2 Laboratory of Experimental Surgery and Regenerative Medicine, Clinic for General Trauma and Reconstructive Surgery, Ludwig-Maximilians University, 80336 Munich, Germany; 3 Center for Applied Tissue Engineering and Regenerative Medicine (Canter), University of Applied Sciences, 80533 Munich, Germany; 4 Dr. Rolf M. Schwiete Research Unit for Osteoarthritis, Orthopedic University Hospital Friedrichsheim gGmbH, 60528 Frankfurt/Main, Germany; 5 Post-Graduate Program in Genetics and Molecular Biology, Federal University of Rio Grande do Sul, 90040-060 Porto Alegre, Brazil; 6 RG Behavioral Biology, Center for Molecular Neurobiology Hamburg (ZMNH), University Medical Center Hamburg-Eppendorf, 20251 Hamburg, Germany; 7 Center for Molecular Neurobiology Hamburg (ZMNH), University Medical Center Hamburg-Eppendorf, 20251 Hamburg, Germany; 8 Department of Genetics, Federal University of Rio Grande do Sul, 90040-060 Porto Alegre, Brazil; 9 Occupational Therapy Faculty, Federal University of Pelotas, 96010-610 Pelotas, Brazil; 10 Pathology Department, Federal University of Rio Grande do Sul, 90040-060 Porto Alegre, Brazil; 11 Post-Graduate Program in Medicine: Medical Sciences, Federal University of Rio Grande do Sul, 90040-060 Porto Alegre, Brazil; 12 Skeletal Research Group, Biosciences Institute, Newcastle University, Newcastle upon Tyne NE1 3BZ, UK; 13 RG Synaptic Wiring and Information Processing, Center for Molecular Neurobiology Hamburg (ZMNH), University Medical Center Hamburg-Eppendorf, 20251 Hamburg, Germany

**Keywords:** MLIII gamma, Lysosomal enzymes, Joints, Extracellular matrix, Cartilage, Tendon

## Abstract

Mucolipidosis type III (MLIII) gamma is a rare inherited lysosomal storage disorder caused by mutations in *GNPTG* encoding the γ-subunit of GlcNAc-1-phosphotransferase, the key enzyme ensuring proper intracellular location of multiple lysosomal enzymes. Patients with MLIII gamma typically present with osteoarthritis and joint stiffness, suggesting cartilage involvement. Using *Gnptg* knockout (*Gnptg^ko^*) mice as a model of the human disease, we showed that missorting of a number of lysosomal enzymes is associated with intracellular accumulation of chondroitin sulfate in *Gnptg^ko^* chondrocytes and their impaired differentiation, as well as with altered microstructure of the cartilage extracellular matrix (ECM). We also demonstrated distinct functional and structural properties of the Achilles tendons isolated from *Gnptg^ko^* and *Gnptab* knock-in (*Gnptab^ki^*) mice, the latter displaying a more severe phenotype resembling mucolipidosis type II (MLII) in humans. Together with comparative analyses of joint mobility in MLII and MLIII patients, these findings provide a basis for better understanding of the molecular reasons leading to joint pathology in these patients. Our data suggest that lack of GlcNAc-1-phosphotransferase activity due to defects in the γ-subunit causes structural changes within the ECM of connective and mechanosensitive tissues, such as cartilage and tendon, and eventually results in functional joint abnormalities typically observed in MLIII gamma patients. This idea was supported by a deficit of the limb motor function in *Gnptg^ko^* mice challenged on a rotarod under fatigue-associated conditions, suggesting that the impaired motor performance of *Gnptg^ko^* mice was caused by fatigue and/or pain at the joint.

This article has an associated First Person interview with the first author of the paper.

## Abstract (precis)

**Summary:** Analyses of *Gnptg* knockout mice indicate that functional joint abnormalities observed in mucolipidosis type III gamma patients are caused by imbalanced metabolism, leading to structural extracellular matrix changes in cartilage and tendons.

## INTRODUCTION

Mucolipidosis type II and III are rare inherited lysosomal storage disorders that are characterized by an extensive clinical spectrum. Mucolipidosis type II (MLII; MIM #252500), the most severe form of the disease, is caused by mutations in the *GNPTAB* gene encoding the membrane-bound precursor of α- and β-subunits of GlcNAc-1-phosphotransferase (EC 2.7.8.17) (Tiede et al., 2005). This enzyme catalyzes the formation of mannose 6-phosphate (M6P) residues on lysosomal enzymes for their proper targeting to lysosomes (Tiede et al., 2005; Kollmann et al., 2010). In patients with MLII, a complete loss of the activity of GlcNAc-1-phosphotransferase results in severe skeletal abnormalities with prenatal or neonatal onset, coarse face features, progressive neurodevelopmental delay, and cardiac and respiratory insufficiency, leading to death in early childhood (Velho et al., 2019). Although biochemically related to MLII, mucolipidosis type III (MLIII) has a later onset of clinical symptoms and a slower disease progression, enabling survival to adulthood. Patients with MLIII can present with mild face coarsening, skin thickening, spinal deformities, tarsal/carpal tunnel syndrome and hip dysplasia, as well as joint stiffness and pain in the shoulders, hips, wrists, knees and/or ankles (Cathey et al., 2010; Oussoren et al., 2018; Tüysüz et al., 2018; Velho et al., 2019). MLIII can be caused by mutations in either *GNPTAB* or *GNPTG*, the latter encoding the γ-subunit of the hexameric (α_2_β_2_γ_2_) GlcNAc-1-phosphotransferase complex (Bao et al., 1996; Raas-Rothschild et al., 2000; Tiede et al., 2005). Based on the affected gene, MLIII is accordingly classified into MLIII alpha/beta (MIM #252600) and MLIII gamma (MIM #252605) (Cathey et al., 2008). In contrast to MLII, in which the activity of GlcNAc-1-phosphotransferase is completely abolished, GlcNAc-1-phosphotransferase displays residual activity in individuals with MLIII alpha/beta and MLIII gamma, which might explain variable clinical presentation among these patients (Velho et al., 2019).

Whereas the majority of lysosomal storage disorders are caused by deficiency of a single lysosomal enzyme, lack of GlcNAc-1-phosphotransferase activity in MLII and MLIII leads to mistargeting of multiple enzymes from the lysosome, provokes their hypersecretion, and thus compromises lysosomal catabolic function in various tissues (Kollmann et al., 2010; Velho et al., 2019). This may result in the accumulation of non-degraded material within lysosomes that eventually impairs cellular homeostasis and imbalances cell metabolism. Previously, we have described a *Gnptg* knockout (*Gnptg^ko^*) mouse model of human MLIII gamma, in which the lysosomal proteome and secretome were remarkably altered due to deficiency of the γ-subunit of GlcNAc-1-phosphotransferase (Di Lorenzo et al., 2018). In particular, we have shown that a subset of lysosomal enzymes involved in the degradation of sulfated glycosaminoglycans (GAGs), including arylsulfatase B (Arsb), β-galactosidase and β-hexosaminidase (Hexb), were mistargeted from the lysosome in *Gnptg^ko^* mouse fibroblasts and secreted into the extracellular space, thus leading to intracellular accumulation of heparan sulfates and chondroitin sulfate (CS)/dermatan sulfate (DS).

Sulfated GAGs represent one of the major components of the extracellular matrix (ECM) and are indispensable for ECM function, especially in the articular cartilage, which provides resistance to compressive and shear forces in the joint. Maintenance of cartilaginous ECM is regulated by a constant turnover of the collagen network and proteoglycans/GAGs by cartilage-resident cells, chondrocytes (Kuettner, 1992; Umlauf et al., 2010; Krishnan and Grodzinsky, 2018). Furthermore, alterations in the GAG sulfation pattern have been linked to aging and degeneration in the articular cartilage (Decker, 2017; Zhang et al., 2019). Because extensive joint pathology, such as limited joint mobility, osteoarthritis and hip joint destruction, is the main clinical complication in patients with MLIII gamma (Velho et al., 2019), it is particularly important to study the role of *Gnptg* in ECM maintenance in the joint.

Using the previously described *Gnptg^ko^* mouse model, we investigated the impact of *Gnptg* deficiency on cartilage development, homeostasis and metabolism, as well as the involvement of lysosomal enzymes in the composition and structure of cartilaginous ECM in mice. Although we did not detect a severe skeletal phenotype in *Gnptg^ko^* mice, we found that lack of *Gnptg* causes significant alterations at both cellular and tissue level. More specifically, we showed (1) hypersecretion of a number of GAG-degrading lysosomal enzymes in *Gnptg^ko^* mouse chondrocytes, (2) altered intracellular morphology of *Gnptg^ko^* chondrocytes due to accumulation of non-degraded GAGs, (3) impaired differentiation of *Gnptg^ko^* chondrocytes, and (4) altered structure of the cartilage ECM in *Gnptg^ko^* mice. Furthermore, as joint stiffness and limited mobility are clinical symptoms observed in both MLII and MLIII patients, we provide comparative data on joint mobility in MLII, MLIII alpha/beta and MLIII gamma patients, which indicate distinct joint pathology in these patients. Finally, by functional and structural analyses of the Achilles tendons isolated from *Gnptab*- and *Gnptg*-deficient mice, we demonstrate that deficiency of either α/β- or γ-subunits of GlcNAc-1-phosphotransferase confers the tendons distinct mechanical properties.

## RESULTS

### Patients with MLIII alpha/beta, MLIII gamma and MLII show differential joint mobility

**Fig. 1.**
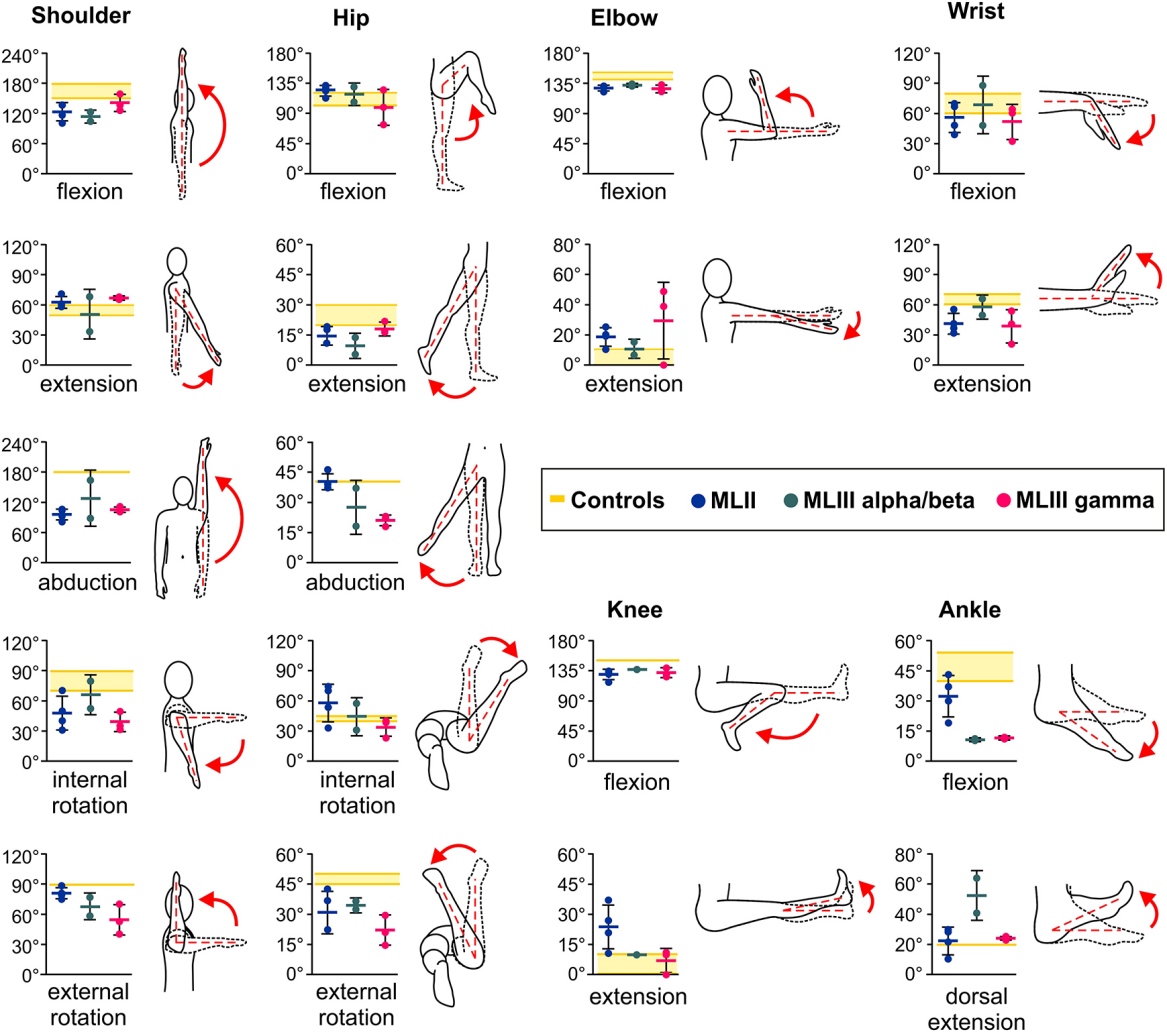
**Mobility characteristics of patients with MLII, MLIII alpha/beta and MLIII gamma.** Range of motions (ROMs, in ^o^) of shoulders, hips, elbows, wrists, knees and ankles were assessed in four patients with MLII, two patients with MLIII alpha/beta and three patients with MLIII gamma using standard goniometric techniques. Values represent the maximum angle and are means±s.d. of three measurements made by the same evaluator. Reference ROMs are indicated in yellow.

Progressive joint stiffness of the hips, shoulders and fingers is a common clinical symptom in patients with MLIII and MLII (Pohl et al., 2010; Tüysüz et al., 2018; Velho et al., 2019). In this study, we performed a comparative analysis of the mobility characteristics of joints in patients with MLIII alpha/beta, MLIII gamma or MLII (Table S1). Using standard goniometric techniques, we assessed the range of motions (ROMs) as a parameter of the movement amplitude at large (shoulders, hips, elbows and knees) and medium (wrists and ankles) joints in four patients with MLII, two patients with MLIII alpha/beta and three patients with MLIII gamma (Fig. 1; Table S2). The analysis revealed differential joint mobility in these patients, with shoulders, hips and ankles being the stiffest joints in MLIII patients. In particular, the MLIII gamma patients presented a significantly decreased ROM during abduction and internal/external rotation of the shoulders, abduction and external rotation of the hips, as well as flexion of the ankles (Fig. 1).

MLII patients presented similar joint mobility defects during shoulder abduction, shoulder internal rotation and hip external rotation, while showing almost normal ankle plantar flexion. In line with the goniometry data on the hip joint mobility in MLIII gamma patients, histological analysis of the femur head articular cartilage from a 43-year-old patient diagnosed with MLIII gamma (Patient 9, Table S1) revealed that the articular surface of the analyzed bone fragment is very irregular and associated with the presence of cracks (or clefts) extending to the deep layer of the cartilaginous tissue (Fig. S1). In addition, pronounced cartilage erosion, chondroblast disorganization and clustering, as well as loss of proteoglycans were observed, whereas the subchondral bone tissue appeared to be preserved. These data suggest that the limited hip joint mobility in MLIII gamma patients could result from pathological alterations in the cartilaginous tissue of the femur head articular surface. Therefore, we next aimed to investigate the role of the γ-subunits of GlcNAc-1-phosphotransferase in cartilage function and homeostasis, using *Gnptg*-deficient mice.

### Lysosomal accumulation of CS is associated with impaired differentiation of primary *Gnptg^ko^* chondrocytes

**Fig. 2.**
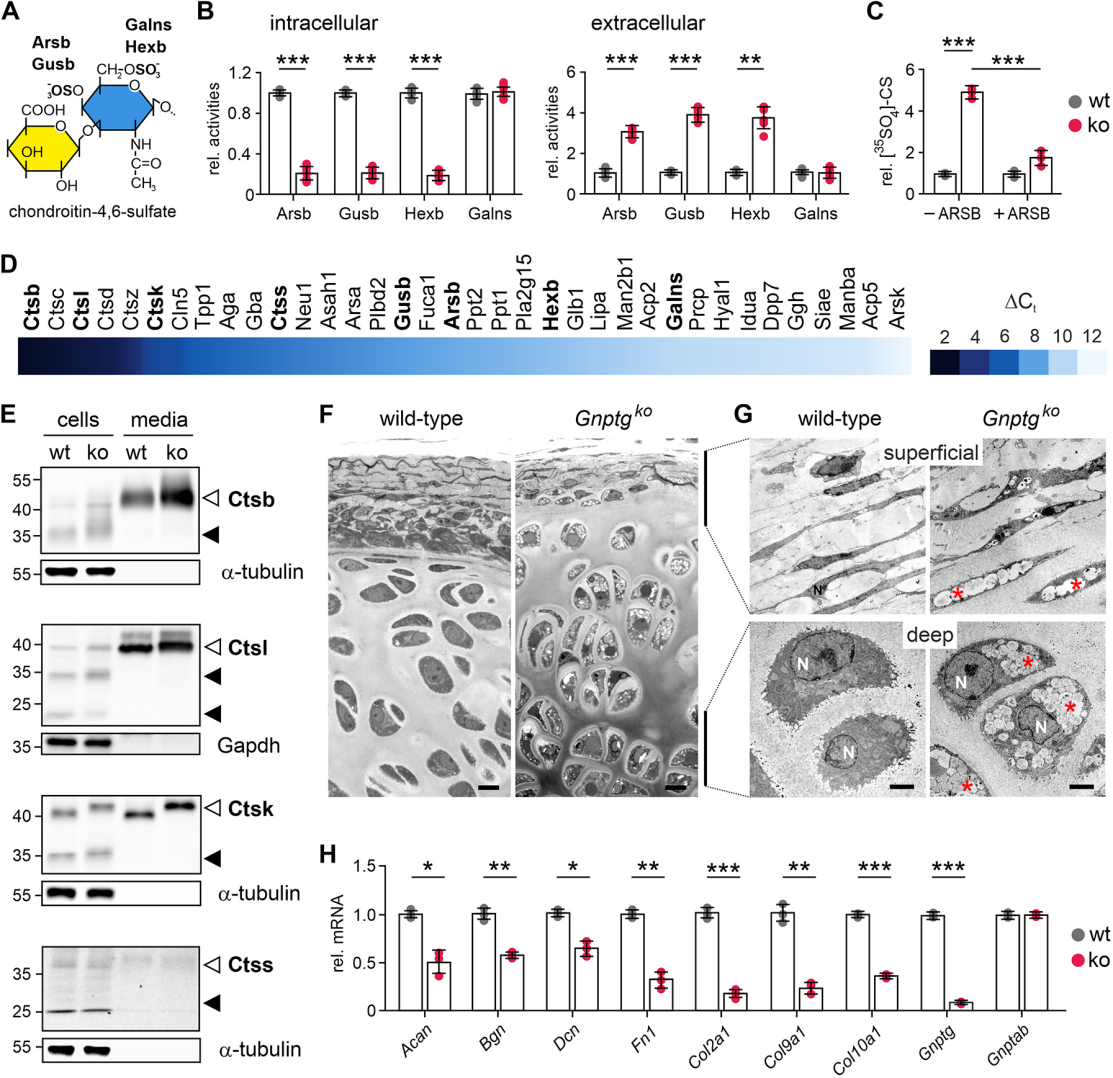
**Hypersecretion of lysosomal enzymes in G*nptg^ko^* ribcage chondrocytes is associated with impaired chondroitin sulfate degradation, chondrocyte differentiation and ECM protein expression.** (A) Schematic overview of lysosomal enzyme degradation of chondroitin-4,6-sulfate, which consists of glucuronate (yellow) and N-acetylgalactosamine (blue). Of note, monosulfated chondroitin-4-sulfate or chondroitin-6-sulfate are also present. The lysosomal glycosidases β-glucuronidase (Gusb) and β-hexosaminidase (Hexb), as well as the lysosomal arylsulfatase B (Arsb) and GalNAc-6-sulfatase (Galns), cleaving C4- or C6-sulfates, respectively, are indicated. (B) Relative intracellular and extracellular enzyme activities of Arsb, Gusb, Hexb and Galns in cell extracts and corresponding media of wild-type (wt) and *Gnptg^ko^* (ko) chondrocytes. Data are shown as means±s.d. generated in at least three independent experiments. **P≤0.01, ***P≤0.001 (unpaired two-tailed Student's *t*-test). (C) Relative total [^35^SO_4_]-chondroitin sulfate (CS) content in wild-type (wt) and *Gnptg^ko^* (ko) chondrocytes after 24 h pulse and 24 h chase in the absence (−) or presence (+) of human recombinant ARSB. Data are shown as means±s.d. from three independent experiments. ***P≤0.001 (unpaired two-tailed Student's *t*-test). (D) Heat map showing the expression (ΔC_t_) of 36 selected genes encoding lysosomal enzymes normalized to *Gapdh* expression*.* Results were obtained by qPCR analysis of wild-type chondrocytes on day 10 of cultivation and represent mean values of three independent experiments (Table S3). Lysosomal enzymes, which were further analyzed here, are marked in bold. (E) Western blot analyses of whole-cell extracts (25 µg protein) and aliquots (20%) of corresponding media of wild-type (wt) and *Gnptg^ko^* (ko) chondrocytes using antibodies against cathepsins B (Ctsb), L (Ctsl), K (Ctsk) and S (Ctss). Representative blots of three independent experiments are shown. Endogenous Gapdh and α-tubulin in cell extracts were used as loading controls. The positions of precursors (open arrowheads) and mature forms (filled arrowheads) of the cathepsins are indicated. (F) Light microscopy of Methylene Blue-stained semi-thin sections (0.5 µm) of ribcage cartilage isolated from 3-week-old wild-type and *Gnptg^ko^* mice. Representative images from two mice per genotype are shown. Scale bars: 10 µm. (G) Representative electron micrographs of the superficial and deep layers of the ribcage cartilage shown in F. Electron-lucent storage material is indicated (red asterisks). N, nuclei. Scale bars: 2 µm. (H) Relative mRNA expression levels of *Acan*, *Bgn*, *Dcn*, *Fn1*, *Col2a1*, *Col9a1*, *Col10a1*, *Gnptg* and *Gnptab* in wild-type (wt) and *Gnptg^ko^* (ko) ribcage chondrocytes. Data are shown as means±s.d. from three independent experiments (Table S4). *P≤0.05, **P≤0.01, ***P≤0.001 (unpaired two-tailed Student's *t*-test).

Recently, we have shown that embryonic fibroblasts from *Gnptg^ko^* mice are characterized by impaired M6P formation on a subgroup of lysosomal enzymes, resulting in their disturbed delivery to lysosomes and subsequent secretion into the extracellular space (Di Lorenzo et al., 2018). Among them are, in particular, lysosomal enzymes involved in the degradation of CS (Fig. 2A). CSs attached to proteoglycans are a predominant type of sulfated GAGs present in cartilaginous ECM produced by chondrocytes (Thorp and Dorfman, 1963), and their constant turnover is essential for the maintenance of cartilage function. Similar to *Gnptg^ko^* fibroblasts, decreased intracellular and increased extracellular activities of Arsb, Hexb and β-glucuronidase (Gusb) were observed in *Gnptg^ko^* chondrocytes, indicating missorting of these lysosomal enzymes in the absence of γ-subunits of GlcNAc-1-phosphotransferase (Fig. 2B). By contrast, lysosomal targeting of GalNAc-6-sulfatase (Galns) was not affected (Fig. 2B). Consistently, the capability for lysosomal degradation of CS was significantly impaired in cells deficient for γ-subunits, as demonstrated by the 5-fold accumulation of [^35^SO_4_]-CS moieties in *Gnptg^ko^* chondrocytes versus wild-type control cells upon metabolic ^35^SO_4_ labeling (Fig. 2C). Importantly, supplementation with human recombinant ARSB led to a significant reduction of the accumulating CS in cultured *Gnptg^ko^* chondrocytes by ∼70% (Fig. 2C). These results show that Arsb plays a crucial role in the degradation and turnover of sulfated GAGs in mouse chondrocytes.

To identify other lysosomal enzymes that are essential for chondrocyte function, we performed a comprehensive quantitative PCR (qPCR) expression analysis of 36 selected genes encoding soluble lysosomal enzymes (Fig. 2D). The transcript levels normalized to *Gapdh* expression varied between ∼2.6 and 11.2, indicating a differential expression of lysosomal enzymes in chondrocytes (Fig. 2D; Table S3). In particular, we detected high mRNA levels of *Ctsb*, *Ctsl*, *Ctsk* and *Ctss*, encoding lysosomal cathepsins involved in the lysosomal proteolysis of endocytosed proteoglycans and collagenous proteins of cartilaginous ECM (Troeberg and Nagase, 2012). Using western blot analysis, we found that lysosomal targeting of these four cathepsins was not affected in *Gnptg^ko^* chondrocytes because the mature, lysosomal forms of the enzymes were present in cell extracts of both wild-type and *Gnptg^ko^* chondrocytes (Fig. 2E). Of note, besides extracellularly secreted matrix metalloproteinases and ADAM/ADAMTS proteases, lysosomal cathepsins can also mediate degradation of ECM proteins (Vizovišek et al., 2019). Accordingly, we observed a pronounced secretion of the precursor forms of cathepsin B, L and K into the cell culture media of wild-type chondrocytes, which was even more elevated in *Gnptg^ko^* cells (Fig. 2E). We therefore assume that proteolytic degradation of endocytosed ECM proteins by cysteine proteases in lysosomes is hardly affected in *Gnptg^ko^* cells; nevertheless, increased secretion of the cathepsins B, L and K by *Gnptg^ko^* chondrocytes could enhance degradation of the ECM extracellularly.

Aiming to assess the impact of *Gnptg* deficiency on morphology and function of chondrocytes, we first analyzed sections of the ribcage cartilage from 3-week-old *Gnptg^ko^* mice by light microscopy. We observed intracellular inclusions inside the ribcage chondrocytes, indicating storage material accumulation, as well as clustering of the cells (Fig. 2F), suggesting aberrant ECM metabolism (Karim et al., 2018). Consistently, by performing ultrastructural analysis of the same tissue region, we identified a remarkable number of enlarged electron-lucent lysosomes, both in chondrocytes of the superficial layer and in hypertrophic chondrocytes of the deep layer in *Gnptg^ko^* ribcage cartilage (Fig. 2G). The observed lysosomal storage material likely represents non-degraded CS, as identified in cultured chondrocytes isolated from *Gnptg^ko^* mice (Fig. 2C). As impaired degradation of proteoglycans and other proteins of cartilaginous ECM can affect the functionality of chondrocytes, we next assessed the expression of cartilage-specific markers in primary *Gnptg^ko^* chondrocytes by reverse transcription qPCR (qRT-PCR). We found that mRNA levels of aggrecan (*Acan*), the most abundant proteoglycan synthesized by chondrocytes, of the proteoglycans biglycan (*Bgn*) and decorin (*Dcn*) and of the structural glycoprotein fibronectin (*Fn1*) were significantly decreased in *Gnptg^ko^* chondrocytes compared to wild-type cells, suggesting that the composition of the cartilage ECM is affected by *Gnptg* deficiency (Fig. 2H; Table S4). In addition, the expression of *Col2a1*, encoding the predominant fibrillar collagen type in proliferating chondrocytes, of *Col10a1*, the marker of differentiated hypertrophic chondrocytes, as well as that of *Col9a1*, encoding fibril-associated collagen type IX, was strongly reduced in *Gnptg^ko^* cells. Importantly, *Gnptab* expression in *Gnptg^ko^* chondrocytes was normal, whereas *Gnptg* transcripts were almost absent in the same cells, indicating that the observed defects were solely associated with deficiency of the γ-subunit of GlcNAc-1-phosphotransferase (Fig. 2H; Table S4).

Taken together, these data demonstrate that the lack of functional γ*-*subunits of GlcNAc-1-phosphotransferase leads to intracellular accumulation of storage material, owing to decreased intracellular activities of CS-degrading lysosomal enzymes as well as to impaired differentiation of *Gnptg^ko^* chondrocytes.

### *Gnptg^ko^* mice display altered ECM homeostasis and compromised chondrocyte function in the cartilage

**Fig. 3.**
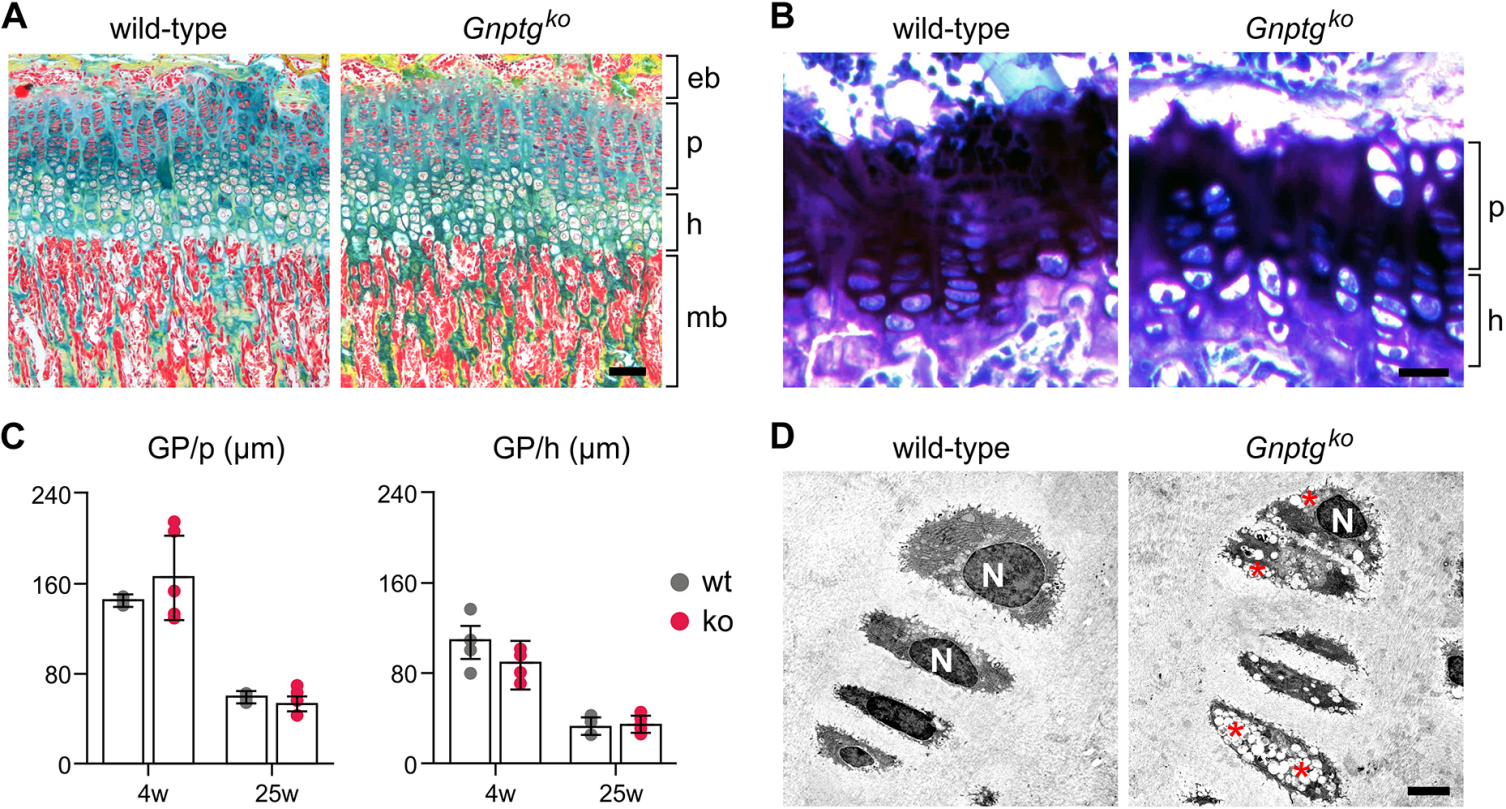
**G*nptg^ko^* mice display unaltered architecture of the growth plate.** (A) Representative Movat pentachrome staining of the growth plate in undecalcified tibia sections from 25-week-old female wild-type and *Gnptg^ko^* mice. eb, epiphysial bone; h, hypertrophic zone; mb, metaphysial bone; p, proliferating zone. Scale bar: 50 µm. (B) Representative Toluidine Blue staining of the growth plate in undecalcified tibia sections from 25-week-old female wild-type and *Gnptg^ko^* mice. h, hypertrophic zone; p, proliferating zone. Scale bar: 20 µm. (C) Quantification of the growth plate (GP) width in proliferating (p) and hypertrophic (h) zones in 4- and 25-week-old wild-type (wt) and *Gnptg^ko^* (ko) mice (*n*=4). (D) Representative electron micrographs of the growth plate hypertrophic chondrocytes in decalcified tibiae cryosections from 25-week-old female wild-type and *Gnptg^ko^* mice. Storage material is indicated (red asterisks). N, nuclei. Scale bar: 2.5 µm.

Chondrocytes in the growth plate play a pivotal role in promoting longitudinal bone growth by endochondral ossification. More specifically, chondrocytes proliferate before differentiating into hypertrophic chondrocytes, which then undergo apoptosis followed by vascularization, resorption of the residual cartilage matrix by osteoclasts, and deposition of new bone by osteoblasts (Karsenty and Wagner, 2002). Based on the above data, showing impaired differentiation of isolated *Gnptg^ko^* chondrocytes *in vitro*, we studied the consequences of the impaired chondrocyte differentiation *in vivo*. Therefore, we performed Movat pentachrome staining of the growth plate in undecalcified tibia sections from 4-week-old wild-type and *Gnptg^ko^* mice. The organization of the growth plate between epiphyseal and metaphyseal bone, as well as the characteristic vertical column architecture of the growth plate chondrocytes, was not disturbed in *Gnptg^ko^* mice (Fig. 3A). Accordingly, by histomorphometric analysis of Toluidine Blue-stained undecalcified tibia sections from 4- and 25-week-old wild-type and *Gnptg^ko^* mice, we did not observe alterations in the thickness of the proliferating and hypertrophic zones of the growth plates (Fig. 3B,C). Finally, contact radiography revealed a moderate growth impairment in 4-week-old *Gnptg^ko^* mice, which was accompanied by a reduced weight gain (Fig. S2). Although no alterations were found in the growth plate architecture and organization, ultrastructural analysis of the growth plate in decalcified tibiae cryosections from 25-week-old *Gnptg^ko^* mice revealed a remarkable number of enlarged electron-lucent lysosomes in hypertrophic chondrocytes (Fig. 3D), similarly to the appearance of chondrocytes in the ribcage cartilage of 3-week-old *Gnptg^ko^* mice (Fig. 2G).

**Fig. 4.**
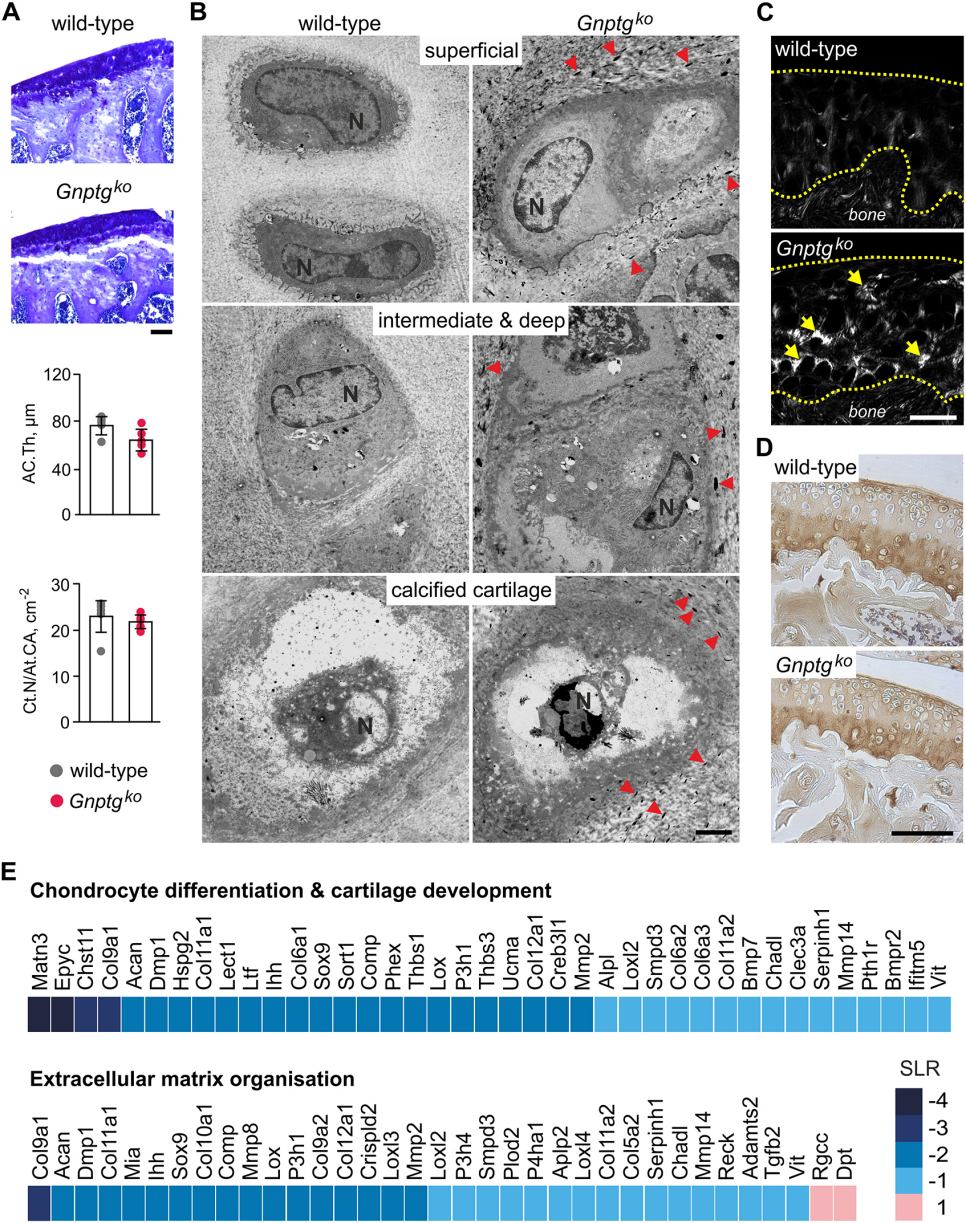
**The articular cartilage in G*nptg^ko^* mice has normal tissue morphology but altered ECM.** (A) Representative Toluidine Blue staining of articular cartilage of undecalcified tibia sections from 40-week-old female wild-type and *Gnptg^ko^* mice. Scale bar: 100 µm. Quantifications of articular cartilage thickness (AC.Th) and chondrocyte number per articular cartilage area (Ct.N/At.CA) of the same mice are given below (mean±s.d., *n*=5). (B) Ultrastructural analysis of chondrocytes in the articular cartilage from 45-week-old female wild-type and *Gnptg^ko^* mice. Representative images of different cartilage layers from two mice per genotype are shown. Red arrowheads indicate electron-dense aggregates in the extracellular matrix of *Gnptg^ko^* mice. Scale bar: 2 µm. (C) Second harmonic generation (SHG) imaging of sagittal sections of the articular cartilage (outlined by dashed yellow lines) from 60-week-old female wild-type and *Gnptg^ko^* mice. Representative images from three mice per genotype are shown. Yellow arrows point to the sites of collagen fibrillation. Scale bar: 50 µm. (D) Immunostaining of sagittal paraffin-embedded sections of the knee joint articular cartilage from 60-week-old wild-type and *Gnptg^ko^* mice against collagen type II. Representative images from three mice per genotype are shown. Scale bar: 100 µm. (E) Heat map generated from microarray data (GEO accession number GSE157180) showing gene expression in the hip joint articular cartilage of 12-week-old female *Gnptg^ko^* mice. The mean values of calculated Signal Log2 Ratio (SLR) relative to wild-type littermate mice are shown for each gene (*n*=6).

Whereas the growth plate cartilage is responsible for longitudinal bone growth, the articular cartilage serves as a load-bearing cushion that minimizes forces bones are subjected to. Similar to an unaltered tissue structure of the growth plate cartilage in 4- and 25-week-old *Gnptg^ko^* mice (Fig. 3), we found no significant changes in the overall tissue morphology of the tibiae articular cartilage in *Gnptg^ko^* mice at 40 weeks of age (Fig. 4). In particular, quantitative analysis of Toluidine Blue-stained tibiae sections revealed no difference in the articular cartilage thickness or in the chondrocyte number between wild-type and *Gnptg^ko^* mice (Fig. 4A). In contrast to the chondrocytes of ribcage or tibia growth plate, we did not detect storage material inside the articular cartilage chondrocytes (Fig. 4B). Nevertheless, the ECM surrounding single chondrocytes appeared to be altered in all zones of the articular cartilage in *Gnptg^ko^* mice, as evidenced by the presence of electron-dense aggregates outside the cells (Fig. 4B). Noteworthy, most of these aggregates had an elongated shape and were aligned along the ECM network surrounding the cells.

Collagen type II is the most abundant fibrillar protein in cartilage and produces a strong second harmonic generation (SHG) signal; thus, alterations in the SHG intensity reflect changes in the collagen structure and organization (Kim et al., 2000). Therefore, to assess changes in the collagen fibrillar network, we performed SHG microscopy of articular cartilage from 60-week-old wild-type and *Gnptg^ko^* mice. In contrast to the low and homogenous SHG signal observed in wild-type animals, we found patches of elevated SHG intensity in the cartilage ECM of *Gnptg^ko^* mice, which occurred in close proximity to the chondrocyte lacunae and indicated collagen structure reorganization (Fig. 4C) (Chen and Broom, 1998; Kim et al., 2000). It should be noted that, despite the stronger SHG signal, the levels of collagen type II in the cartilage of *Gnptg^ko^* mice were unaltered relative to those in wild-type animals, as demonstrated by immunohistochemistry of the knee joint tissues (Fig. 4D). Thus, the elevated SHG signal in the cartilage of *Gnptg^ko^* mice likely reflects structural changes in the collagen type II fibrillar network rather than its increased abundance.

To gain more information on the molecular mechanisms leading to alterations in the cartilage ECM in *Gnptg*-deficient mice, we performed gene expression profiling of the hip joint articular cartilage from 12-week-old wild-type and *Gnptg^ko^* mice. Gene ontology enrichment analysis of the obtained microarray data revealed decreased expression of a number of genes involved in chondrocyte differentiation, cartilage development and ECM organization. Among those, matrilin-3 (*Matn3*) and epiphycan (*Epyc*), which are essential components of collagen fibrillar network in cartilage (Klatt et al., 2011; Brachvogel et al., 2013), were the most downregulated in *Gnptg^ko^* mice, with a Log2 ratio of −4 compared to wild-type animals (Fig. 4E). Genes encoding other collagen fibril-associated proteins, such as collagen type IX (*Col9a1*), cartilage oligomeric matrix protein (*Comp*) and thrombospondin-1 (*Thbs1*), were found to be moderately downregulated (Log2 ratios between −2 and −3). Although we could not detect significant alterations in the protein amounts of collagen type IX, COMP, Matn3 and Thbs1 in the articular cartilage of *Gnptg^ko^* mice (Fig. S3) at the age of 60 weeks, we suppose that cartilaginous ECM homeostasis can be affected in *Gnptg^ko^* mice by transcriptional downregulation of these genes at a younger age. In line with the gene expression data in ribcage chondrocytes (Fig. 2H), the hypertrophic chondrocyte markers *Acan* and *Col10a1*, as well as the transcription factor *Sox9*, were strongly downregulated in *Gnptg^ko^* mice, suggesting impaired chondrocyte differentiation. Based on these data, we conclude that *Gnptg* deficiency compromises both ECM homeostasis and chondrocyte function in mouse cartilage and may thus affect overall stability and movement of the joint.

### Structural and functional analysis of the Achilles tendon in *Gnptg^ko^* mice reveals an age-dependent reduction in the stiffness of collagen fibrils

**Fig. 5.**
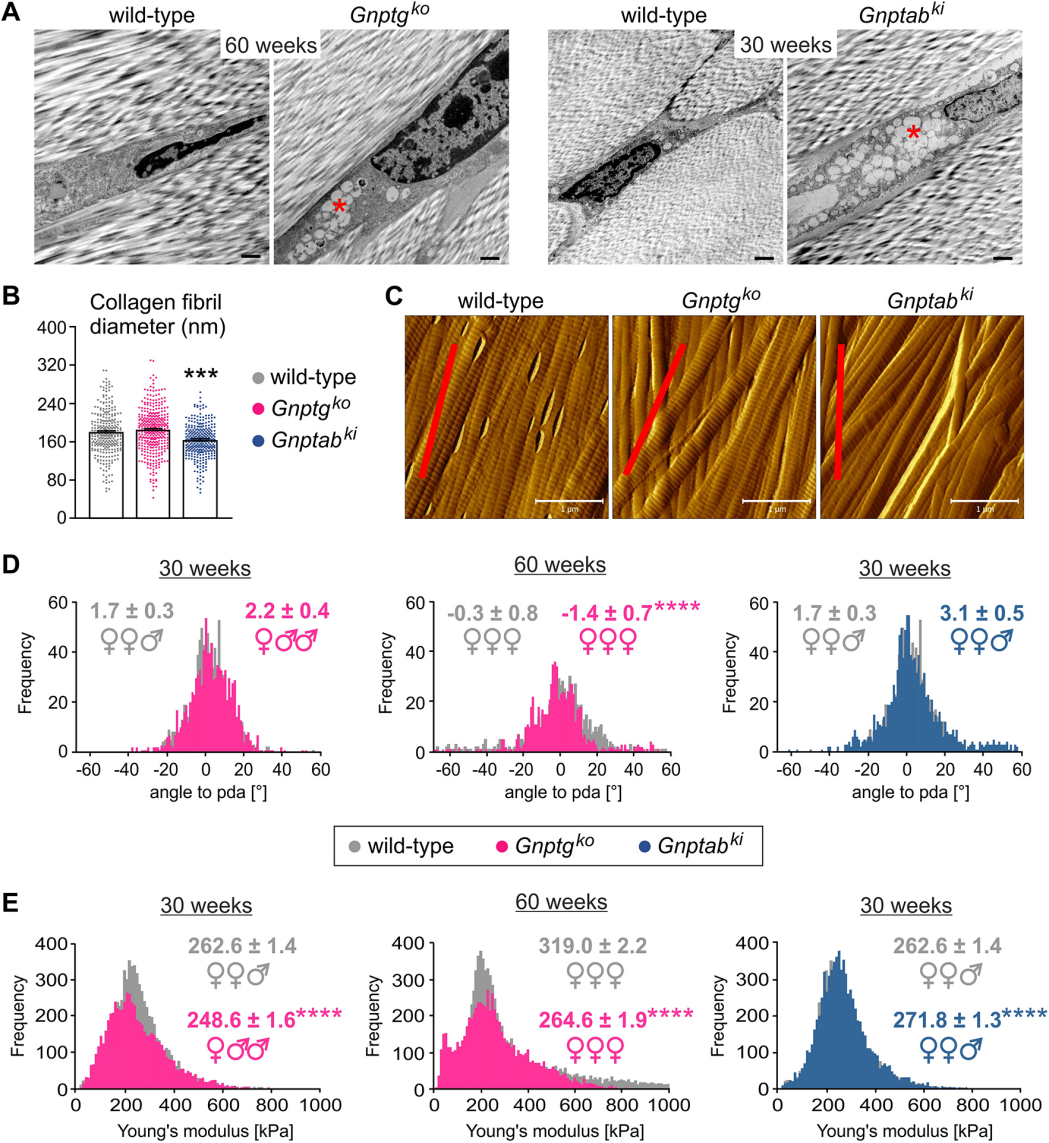
**Structural and functional analysis of the Achilles tendon in *Gnptab^ki^* and *Gnptg^ko^* mice.** (A) Ultrastructural analysis of the Achilles tendon from *Gnptg^ko^* (60-week-old), *Gnptab^ki^* (30-week-old) and age-matched wild-type mice. Representative images from two mice per genotype are shown. Electron-lucent storage material is indicated (red asterisks). Scale bars: 1 µm. (B-E) Atomic force microscopy (AFM) analysis of collagen type I fibrils in the Achilles tendons of 30- and 60-week-old mice. The results obtained from three mice per genotype per age are shown (sex of each mouse is indicated as ♂ or ♀). (B) Average collagen fibril diameters in 30-week-old mice (mean±s.e.m.). Each dot represents a single fibril measured. ***P≤0.0001 (unpaired two-tailed Student's *t*-test). (C) Representative AFM topographical images of collagen type I fibrils of 30-week-old mice. The proximodistal axis (pda) is shown as a red line. Scale bars: 1 µm. (D) Frequency distribution of angles to pda for single collagen fibrils in 30- and 60-week-old mice. Values shown are means±s.e.m. ****P≤0.0001 (two-tailed Mann–Whitney test). (E) Frequency distribution of Young's moduli denoting the elastic properties of collagen fibrils in 30- and 60-week-old mice. Values shown are means±s.e.m. ****P≤0.0001 (two-tailed Mann–Whitney test).

Movement at the joint occurs owing to contracting muscles that transmit forces onto bones through collagenous connective tissues, called tendons. The Achilles tendon is the strongest and largest tendon in the human body that, acting together with the shin muscles, controls plantar flexion of the ankle joint. Because patients with MLIII, but not MLII, displayed impaired plantar flexion in their ankles (Fig. 1), we performed structural and functional analyses of the Achilles tendons isolated from 30- and 60-week-old *Gnptg^ko^* and 30-week-old *Gnptab^ki^* mice. By electron microscopy of the Achilles tendons, we found enlarged lysosomal compartments in tendon cells (tenocytes) of both mutant mouse models, which were filled with electron-lucent storage material (Fig. 5A), indicating compromised cell metabolism and matrix turnover in the tendon.

The ECM of tendons consists of collagen fibrils that align laterally to form fibril bundles and fibers, the structure and composition of which define their mechanical properties (Zhang et al., 2005). Therefore, by means of atomic force microscopy (AFM), we measured the diameter of single collagen fibrils in tendons of 30-week-old *Gnptg^ko^*, *Gnptab^ki^* and wild-type mice. The tendon fibril thickness in *Gnptg^ko^* mice was comparable to that in wild-type controls (∼170 nm), whereas the tendons from *Gnptab^ki^* mice were composed of fibrils of a significantly decreased diameter (∼150 nm; Fig. 5B). Furthermore, using the calculated mean angle between collagen fibrils and the proximodistal axis of the fiber as a measure of fibril alignment, we found that fibrils in the tendons of 30-week-old *Gnptg^ko^* and *Gnptab^ki^* mice align normally, whereas 60-week-old *Gnptg^ko^* mice display a modest but significant fibril misalignment in their tendons compared to age-matched wild-type mice (−1.4±0.7° in *Gnptg^ko^* versus −0.3±0.8° in wild-type mice) (Fig. 5C,D). Finally, by means of indentation-type (IT) AFM, we analyzed the elastic modulus of collagen fibrils of the Achilles tendons from *Gnptg^ko^* and *Gnptab^ki^* mice, which is defined as Young's modulus (Fig. 5E). Significantly increased Young's modulus values were observed in 30-week-old *Gnptab^ki^* mice compared to age-matched wild-type mice (271.8±1.3 kPa in *Gnptab^ki^* versus 262.6±1.4 kPa in wild-type mice), indicating elevated tendon stiffness in the mutant mice. By contrast, stiffness of collagen fibrils in *Gnptg^ko^* mice at 30 weeks of age was decreased, as demonstrated by significantly reduced Young's modulus values (248.6±1.6 kPa in *Gnptg^ko^* versus 262.6±1.4 kPa in wild-type mice), with the difference from that in wild-type mice being even more pronounced at 60 weeks of age (264.6.8±1.9 kPa in *Gnptg^ko^* versus 319.0±2.2 kPa in wild-type mice). Altogether, these data suggest that defects in either *Gnptg* or *Gnptab* define differential mechanical properties of the Achilles tendon in mice, resulting in lower tendon stiffness in *Gnptg^ko^* mice or increased tendon stiffness in *Gnptab^ki^* mice.

### Age-dependent motor impairment in *Gnptg^ko^* mice

**Fig. 6.**
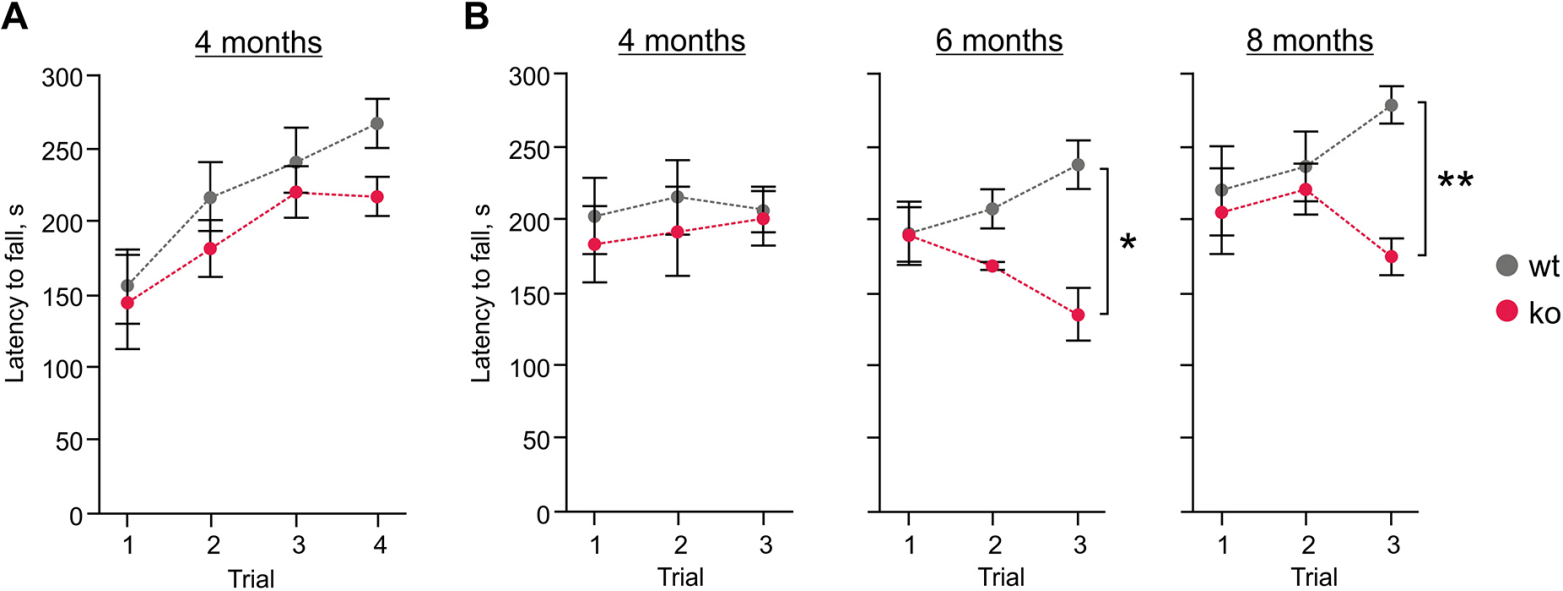
***Gnptg^ko^* mice display age-dependent impaired motor performance on a rotating rod.** (A,B) *Gnptg^ko^* (ko) and wild-type (wt) male mice were tested on a rod rotating at an accelerating speed in four consecutive trials (A) or at a constant speed in three consecutive trials (B). Values are shown as means±s.e.m. (*n*=10 for wild-type, *n*=7 for *Gnptg^ko^*). *P<0.05, **P<0.01 (Sidak's multiple comparisons following a mixed two-way ANOVA).

Previous studies have reported behavioral deficits, in particular in motor function, in mouse models of MLII and MLIII (Idol et al., 2014; Paton et al., 2014). We thus assessed motor function in 4-, 6- and 8-month-old *Gnptg^ko^* and wild-type mice in the rotarod test using two different protocols. In the accelerated rotarod test, the performance of *Gnptg^ko^* mice did not differ from that of wild-type mice, suggesting that coordination and motor learning, and thereby cerebellar function, are normal in *Gnptg^ko^* mice (Fig. 6A). We then used a protocol that challenges motor performance by forcing the mice to run at a high rotation speed in three consecutive trials within a daily session. In this case, the mixed two-way ANOVA detected a significant effect of the interaction between genotype and trial in mice aged 6 (*F*_2,30_=9.665; *P=*0.0006) and 8 (*F*_2,30_=2.713; *P=*0.0826) months, but not in those aged 4 months (*F*_2,30_=0.137; *P=*0.8724). Post hoc comparisons showed that 6- and 8-month-old *Gnptg^ko^* mice fell faster from the rod than the age-matched wild-type mice, specifically in the third trial of each session, suggesting that their impaired motor performance was caused by fatigue or pain (Fig. 6B). Noteworthy, there were no differences between the genotypes in the grip strength test performed in 5- and 7-month-old mice, suggesting that the impaired performance of *Gnptg^ko^* mice in the rotarod test was not due to impaired muscular function (Fig. S4A). In contrast to the mild neurological deficits of *Gnptg^−/−^* male mice reported previously (Idol et al., 2014), we did not detect any abnormal behavior in our *Gnptg^ko^* male mice at similar ages. Specifically, the open field test did not reveal differences in the performance of 4- and 6-month-old *Gnptg^ko^* and wild-type mice, indicating that general locomotion and novelty-induced exploration and anxiety are not affected in *Gnptg^ko^* mice (Fig. S4B,C).

## DISCUSSION

Formation of M6P moieties by GlcNAc-1-phosphotransferase confers lysosomal enzymes a targeting signal that ensures their delivery to lysosomes and proper lysosomal function (Tiede et al., 2005; Kollmann et al., 2010). Accordingly, lack of GlcNAc-1-phosphotransferase activity due to defects in the α-, β- or γ-subunits compromises lysosomal catabolic function in the cell and can eventually cause MLII, MLIII alpha/beta or MLIII gamma in humans (Velho et al., 2019). In MLII and MLIII patients, clinical symptoms are generally broad, but skeletal abnormalities are common. Whereas MLII results in severe skeletal defects and is recognizable at birth, MLIII alpha/beta and MLIII gamma patients have much milder forms of skeletal pathology, such as joint stiffness, osteoarthritis and hip joint destruction, and are clinically indistinguishable. Because MLIII gamma is often misdiagnosed and not verified by genetic analyses, there is a lack of correlation between the clinical course of MLIII gamma and mutations in *GNPTG*, encoding the γ-subunit of GlcNAc-1-phosphotransferase (Velho et al., 2019). The present study provides new insights into the role of the γ-subunit in coupling lysosomal homeostasis with skeletal function, including chondrocyte differentiation, cartilage maintenance, as well as the structure of cartilaginous and tendinous ECM, using a mouse model of MLIII gamma.

In *Gnptg^ko^* embryonic fibroblasts, impaired M6P formation on a subset of GAG-degrading lysosomal enzymes results in the secretion of these enzymes into the extracellular space, while bypassing lysosomes (Di Lorenzo et al., 2018). CS is the major type of sulfated GAGs synthesized by chondrocytes and its turnover is essential for cartilage maintenance (Thorp and Dorfman, 1963). Our data suggest that *Gnptg* deficiency not only disturbs lysosomal homeostasis through hypersecretion of the CS-degrading lysosomal enzymes Arsb, Hexb and Gusb, but also affects the chondrocyte program for differentiation and ECM synthesis. Secretion of the lysosomal cysteine proteases cathepsin B, L and K appeared to be more pronounced in *Gnptg^ko^* chondrocytes. In addition to their intracellular function, these proteases also act extracellularly, by cleaving a number of ECM components including the glycoproteins laminin and fibronectin, as well as collagens (Buck et al., 1992; Mort et al., 2016; Vidak et al., 2019). Therefore, hypersecretion of these proteases by chondrocytes could result in increased degradation of cartilaginous ECM and compromised cartilage homeostasis in *Gnptg*-deficient mice. Cartilage possesses limited self-regenerative capacity, and the efficiency of cartilage repair depends on the composition and structure of ECM, which delivers specific biological signals to the embedded chondrocytes (Emans and Peterson, 2014; Mao et al., 2019). Hence, even minor changes in the cartilage ECM can affect chondrocyte differentiation and provide a positive feedback that aggravates cartilage dysfunction.

Although cultured *Gnptg^ko^* ribcage chondrocytes exhibited decreased intracellular activity of Arsb and elevated accumulation of CS, the addition of recombinant human ARSB (Naglazyme^®^) to the culture medium resulted in significant restoration of CS degradation. These data support our previous findings in *Gnptg^ko^* fibroblasts (Di Lorenzo et al., 2018) and point to a critical role of Arsb in the turnover of CS by chondrocytes. In fact, Arsb is a key lysosomal enzyme regulating skeletal remodeling, and its deficiency in mice results in growth retardation and lysosomal storage in both bone cells and chondrocytes (Pohl et al., 2018). Treatment of mouse *Arsb^m/m^* chondrocytes with Naglazyme^®^ *in vitro* has been shown to significantly reduce lysosomal defects, despite a relatively low uptake of the recombinant ARSB by chondrocytes (Hendrickx et al., 2020). However, cartilage deficits could not be corrected in *Arsb^m/m^* mice receiving Naglazyme^®^, probably due to poor delivery of the systemically administered ARSB into chondrocytes (Hendrickx et al., 2020). Although it is tempting to recognize recombinant ARSB as a potential therapeutic for correcting cartilage defects caused by abnormal GAG accumulation in MLIII gamma, the issue of poor targeted delivery of ARSB to chondrocytes needs to be considered in future *in vivo* studies.

Short stature and abnormal bone development (dysostosis multiplex) are typically recognized in MLIII patients, suggesting growth plate involvement (Cathey et al., 2010; Oussoren et al., 2018; Tüysüz et al., 2018). Although storage material was found to accumulate in the growth plate chondrocytes from *Gnptg^ko^* mice, the morphology of the growth plate was normal, while skeletal growth was only moderately retarded in *Gnptg*-deficient mice. Despite the lack of obvious skeletal defects in *Gnptg^ko^* mice, in contrast to MLIII patients, which can be explained by distinct posture and body weight distribution in mice and humans, *Gnptg^ko^* mice displayed remarkable alterations in the articular cartilage ECM. The articular cartilage endures continuous mechanical stress during load bearing, thus protecting the bone. This is accomplished by a collagen fibrillar network that confers the ECM tensile properties, while proteoglycans (e.g. the most abundant aggrecan) endow it with compression stiffness (Poole et al., 2001). One of the earliest features of articular cartilage damage, e.g. in osteoarthritis, is softening of the tissue, which is associated with radial alignment of collagen fibrils in the middle and deep layers of the cartilage, even when no macroscopic tissue damage can be observed (Broom, 1982; Chen and Broom, 1998; Hosseini et al., 2013). In striking accordance with this, *Gnptg^ko^* mice demonstrated no morphological changes in the articular cartilage but displayed significant fibrillation within the tissue, as evidenced by radially aligned SHG-positive structures. The enhanced SHG signal in *Gnptg^ko^* mice likely arises from structural changes in the collagenous network that is formed by collagen type II, as a dominant fibril-forming collagen in the cartilage, and minor collagen types IX and XI, maintaining integrity of the fibrils and allowing the formation of collagen networks (Eyre and Wu, 1995; Wu and Eyre, 1995; Chen and Broom, 1998). Supporting this idea, we found *Col9a1* and *Col11a1* to be downregulated in the articular cartilage of *Gnptg^ko^* mice, while collagen type II abundance was not altered. In addition, *Gnptg^ko^* mice displayed a significantly impaired limb motor function, this defect becoming more obvious with age and suggesting that the deficient motor function in *Gnptg^ko^* mice is associated with fatigue and/or pain at the joint. Importantly, our findings in *Gnptg^ko^* mice are consistent with the limited mobility of hips, shoulders and ankles, as well as with severe osteoarthritic destruction of the femoral head cartilage, that we observed in MLIII gamma patients. Based on these data, we postulate that deficiency of the γ-subunit of GlcNAc-1-phosphotransferase causes structural changes in the fibrillar network of articular cartilage ECM, which likely precede the osteoarthritic damage typically observed in patients with MLIII gamma.

In contrast to most of the lysosomal storage diseases, lack of GlcNAc-1-phosphotransferase activity in MLII and MLIII compromises the activities of multiple lysosomal enzymes, resulting in a plethora of molecular and pathological events in various tissues (Kollmann et al., 2010; Velho et al., 2019). Because physical movement at the joint is accomplished by the coordinated action of muscles, tendons and ligaments, one can expect a complex involvement of these connective tissues into the impaired joint mobility in patients with MLII and MLIII. In this study, we found that deficiency of either the α/β- or γ-subunits of GlcNAc-1-phosphotransferase confers the Achilles tendons isolated from *Gnptab^ki^* and *Gnptg^ko^* mice distinct mechanical properties. Specifically, collagen fibrils of the tendons of *Gnptab^ki^* mice were significantly decreased in diameter, suggesting that increased tendon stiffness in *Gnptab*-deficient mice results from disorganized tendinous ECM rather than from tendon hypertrophy (Franchi et al., 2013; Wiesinger et al., 2015). In contrast, tendons from *Gnptg^ko^* mice were more compliant, with no altered thickness of collagen fibrils observed. Akin to other mechanosensitive tissues, mechanical properties and structure of tendons are defined by changes in ECM organization, as well as in gene expression, occurring in tenocytes in response to mechanical stimuli (Nourissat et al., 2015; Wiesinger et al., 2015). Accordingly, accumulation of storage material that we observed in tenocytes of *Gnptab^ki^* and *Gnptg^ko^* mice suggests that altered cellular metabolism and ECM turnover might affect tendon remodeling and overall joint function in the mutant mice. This study is the first to propose the contribution of tendons to the MLII and MLIII joint pathology. However, it remains unclear how deficiency of either the α/β- or γ-subunits causes distinct adaptive responses of tenocytes to a mechanical strain and leads to differential collagen fibril organization in the mouse tendon. Future studies will examine the effects of different mechanical strains on the structural and functional properties of tendons and joints in *Gnptab^ki^* and *Gnptg^ko^* mice.

In conclusion, our data suggest that lack of GlcNAc-1-phosphotransferase activity resulting from defects in the γ-subunits is causative of structural changes in the ECM of connective and mechanosensitive tissues, such as cartilage and tendons, resulting in functional abnormalities of the joint. In mice, *Gnptg* deficiency affects cartilage function both intracellularly, through imbalanced lysosomal homeostasis, and extracellularly, via an altered ECM that can disturb chondrocyte differentiation. Although joint cartilage destruction and stiffness have been recognized as hallmarks of MLIII gamma, the exact molecular mechanisms underlying these defects are poorly understood. The activity of GlcNAc-1-phosphotransferase is critical in disease progression and severity, yet additional molecular factors might be involved (Velho et al., 2019). Therefore, it remains to be investigated whether joint abnormalities in MLIII gamma are solely caused by a comprehensive lysosomal dysfunction owing to reduced GlcNAc-1-phosphotransferase activity, and thus can be corrected by respective lysosomal enzyme replacement therapies, or the defects result from a disturbed, yet undefined, function of the γ-subunit as well.

## MATERIALS AND METHODS

### Antibodies

Western blot analyses were performed using the following antibodies: mouse monoclonal anti-cathepsin K (sc-48353; 1:1000), goat polyclonal anti-cathepsin S (sc-6503; 1:500) and rabbit polyclonal anti-Gapdh (sc-25778;1:2000) antibodies from Santa Cruz Biotechnology; goat polyclonal anti-cathepsin B antibody (GT15047; 1:1000) from Neuromics; goat polyclonal anti-cathepsin L antibody (AF1515; 1:500) from R&D Systems; mouse monoclonal anti-α-tubulin antibody (T9026; 1:1000) from Sigma-Aldrich. For immunohistochemistry, the following antibodies were used: mouse anti-collagen type II (CP18, Merck; 1:500), rabbit anti-collagen type IX [1:3000; self-made (Budde et al., 2005)], rabbit anti-cartilage oligomeric matrix protein 4-1 (COMP) [1:500; self-made (Mayorca-Guiliani et al., 2019)], rabbit anti-Matn3 [1:500; self-made (Klatt et al., 2000)] and mouse anti-Thbs1 (BA24, Calbiochem; 1:200).

### Animals

*Gnptg^ko^* (C57Bl/6) and *Gnptab^ki^* (C57Bl/6–129/SvJ, 50:50) mice have been described previously (Kollmann et al., 2012, 2013; Di Lorenzo et al., 2018). We generally analyzed female littermate mice from heterozygous matings, unless stated differently. All mice were kept in a pathogen-free environment with a 12-h light/dark cycle, 45% to 65% relative humidity and 20°C to 24°C ambient temperature, in open or individually ventilated cages with wood shavings bedding and nesting material, in groups not surpassing six animals. The mice had access to tap water and standard rodent chow *ad libitum*. All animal experiments were approved by the animal facility of the University Medical Center Hamburg-Eppendorf and by Behörde für Gesundheit und Verbraucherschutz. The care and use of experimental animals complied with all relevant local animal welfare laws, guidelines and policies.

### Skeletal analysis

After sacrifice, the dissected skeletons were fixed in 3.7% phosphate buffered saline (PBS)-buffered formaldehyde for 18 h at 4°C and then stored in 80% ethanol. All skeletons were first analyzed by contact radiography using a Faxitron X-ray cabinet (Faxitron Xray) to measure the length of the lumbar spine, femora and tibia. For undecalcified bone histology, tibiae were dehydrated in ascending alcohol concentrations and then embedded in methylmetacrylate as described previously (Schulze et al., 2011). Sections of 4 μm thickness were cut in the sagittal plane on a Microtec rotation microtome (Techno-Med). Sections were stained following a standard protocol for Toluidine Blue staining, and histomorphometry was performed according to the American Society for Bone and Mineral Research (ASBMR) guidelines (Parfitt et al., 1987) using the OsteoMeasure histomorphometry system (Osteometrics). Movat pentachrome staining (Morphisto) was carried out according to the manufacturer's instructions.

### Histological analysis of cartilage

For cartilage ECM protein staining, the hind legs were washed in PBS for 24 h and decalcified in 10% tris-ethylenediaminetetraacetic acid (EDTA) for 10-14 days. Decalcified limbs were further processed and embedded in paraffin in sagittal orientation. Serial sections were taken at a thickness of 8 µm. Joint sections were stained with antibodies against collagen type II, collagen type IX, COMP, Matn3 and Thbs1. For collagen type II staining, the sections were initially digested using pepsin (0.025% in 0.2 M HCl; Merck) for 15 min at 37°C. An enzymatic digestion with hyaluronidase (500 U/ml in hyaluronidase buffer pH 5.0; Merck) for 30 min at 37°C and proteinase K (10 µg/ml in proteinase buffer pH 7.4; Merck) for 10 min at 55°C was conducted for all the antibodies to demask the antigens. After quenching the endogenous peroxidase with 3% H_2_O_2_ for 10 min and blocking with Zytomed blocking solution (Zytomed Systems) for 5 min at room temperature, the sections were incubated with respective primary antibodies overnight at 4°C. ZytoChem Plus HRP-polymer anti-rabbit or anti-mouse secondary antibodies (Zytomed Systems) and 3,3′-diaminobenzidine (0.05% DAB and 0.015% H_2_O_2_ in 0.01 M PBS pH 7.2; Merck) were used to detect the specific primary antibody bindings. The brownish staining through the oxidation of the DAB was complemented with a nuclear counterstaining with Mayer's Hematoxylin (Merck) for 10 s at room temperature.

### Isolation and culture of primary chondrocytes

Chondrocyte progenitor cells were isolated from 10-day-old *Gnptg^ko^* or wild-type mice. Sterna from four to five mice per genotype were collected, and the cells were separated by digesting the tissue in 0.1% collagenase Ia solution followed by 0.2% collagenase Ia solution. The isolated cells were then cultured in Dulbecco's modified eagle medium/Ham's F-12 (1:1) medium supplemented with 10% fetal calf serum (Biochrom AG). At a total cell confluence of 80%, chondrocyte differentiation was induced by the addition of ascorbic acid (50 µg/ml) and culture for 10 days.

### Transcriptome analysis

To yield enough material for gene array, six wild-type and six *Gnptg^ko^* mice were sacrificed at 12 weeks of age, hip joints were dissected, and cartilage caps were isolated and placed on dry ice. Using a mortar and pestle, the cartilage samples were then crushed into a fine powder in liquid nitrogen and subjected to RNA isolation. Total RNAs were extracted using PEQ Gold Total RNA Isolation Kit (VWR) according to the manufacturer's instructions. RNA quality was assessed by photospectrometry (NanoDrop 1000, Thermo Fisher Scientific) and integrity analysis (Tapestation 2200, Agilent Technologies). The genome-wide gene expression analysis was performed using the Clariom D mouse system (Thermo Fisher Scientific) according to the manufacturer's GeneChip™ WT PLUS reagent kit manual. Briefly, 100 ng of total RNA samples pooled from six mice per genotype were used for the synthesis of second-cycle single-stranded complementary DNA (cDNA). Subsequently, 5.5 µg of fragmented and labeled cDNA were used for gene chip hybridization. After washing and staining with the Affymetrix Fluidics Station 450, microarrays were scanned with the Affymetrix Gene Chip Scanner 7G, and the signals were analyzed with the Transcriptome Analysis Console software (TAC 4.0, Thermo Fisher Scientific) using default analysis settings (version 2) and Gene+Exon –SST-RMA (Signal Space Transformation-Robust Multi-Chip Analysis) as summarization. For gene ontology enrichment analysis, differentially expressed genes (absolute 1.5 SLR and adjusted *P*-value≤0.05) were used to find enriched gene ontology biological processes using GOseq (Young et al., 2010). Gene ontology terms with an adjusted *P*-value≤0.05 were regarded as statistically significant. For quantitative mRNA expression analysis, RNA isolation from cultured cells, cDNA synthesis and quantitative real-time PCR using pre-designed Taqman-Assays (Thermo Fisher Scientific) were performed as previously described (Di Lorenzo et al., 2018). Relative mRNA expression levels of analyzed genes were normalized to the level of *Gapdh* mRNA in the same sample using the comparative CT method (2^–ΔΔCT^).

### Protein analysis

Primary chondrocytes were cultured in serum-free Opti-MEM™ medium (Gibco) for 24 h before the cells and conditioned media were collected. Media were concentrated 4-fold using Amicon Ultra-0.5 ml (3 kDa cutoff) centrifugal filters (Merck). Cells were lysed in PBS containing 0.5% Triton X-100 and protease inhibitors for 30 min at 4°C. Lysates were clarified by centrifugation at 16,000 ***g***, and the protein content in supernatants was measured using the Roti^®^quant Protein Assay (Carl Roth). Cell extracts and concentrated conditioned media were solubilized in reducing Laemmli sample buffer for 5 min at 95°C, separated by sodium dodecyl sulphate-polyacrylamide gel electrophoresis (SDS-PAGE) and transferred to nitrocellulose membranes. Membranes were blocked in Tris-buffered saline (TBS; pH 7.4) containing 0.05% Tween 20 and 5% milk powder or 1% bovine serum albumin for 1 h, and then incubated with the respective primary antibodies in blocking buffer for 1 h or overnight at 4°C. Blots were washed with TBS containing 0.05% Tween 20 and incubated for 1 h at room temperature with appropriate horseradish peroxidase-coupled secondary antibodies (Dianova) diluted in blocking buffer. Membranes were washed, and immunoreactive protein bands were visualized by chemiluminescence. The enzymatic activities of lysosomal enzymes in protein extracts of primary cultured cells and corresponding conditioned media were assayed using appropriate 4-nitrophenol- or 4-methylumbelliferone-based substrates (Kollmann et al., 2012; Di Lorenzo et al., 2018).

### GAG analysis

For GAG analysis, primary chondrocytes were incubated for 24 h in serum-free Opti-MEM™ medium containing 100 µCi/ml Na_2_^35^SO_4_ (Hartmann Analytic). Cells were then washed twice with PBS and incubated for 24 h with serum-free Opti-MEM™ medium in the presence or absence of 10 µg/ml human recombinant ARSB (Naglazyme^®^, BioMarin). GAGs were isolated from cell lysates using DEAE-Sepharose (Sigma-Aldrich) anion exchange chromatography and subjected to digestion with heparinase I, II and III kindly provided by Dr J. Esko, University of California, San Diego, CA, USA (Lawrence et al., 2012). The treatment-resistant CS/DS were purified and radioactivity quantified by liquid scintillation counting (Lamanna et al., 2011).

### Electron microscopy

Tibiae were fixed with 4% paraformaldehyde and 1% glutaraldehyde in 0.1 M phosphate buffer (pH 7.4) overnight and decalcified for 3-4 weeks in 10% EDTA. Thereafter, 100 µm thick sections were prepared with a vibratome and post-fixed in 1% OsO_4_, dehydrated and embedded in Epon. Ultrathin sections (60 nm) were cut and mounted on copper grids. Sections were stained using uranyl acetate and lead citrate.

### SHG microscopy

For SHG analysis of articular cartilage, undecalcified knee joint samples from 60-week-old wild-type and *Gnptg^ko^* mice were dehydrated in ascending alcohol concentrations and embedded into methylmetacrylate. Sections of 4 μm thickness were cut in the sagittal plane on the microtome and then mounted in Poly-Mount™ mounting medium (Polysciences). SHG imaging was carried out using a multiphoton scanning microscope (DF-scope from Sutter Instrument), custom modified by Rapp OptoElectronic and controlled by ScanImage 2017b (Vidrio Technologies). Second harmonics were excited with an ytterbium-doped 1070-nm fiber laser (Fidelity-2, Coherent), which was focused on the sample using a 25× water immersion objective (Leica HC FLUOTAR L 25x/0.95 W VISIR, Leica). The laser power in the focal plane was 110 mW. The forward-propagating green SHG signal was collected through an oil immersion condenser (numerical aperture 1.4, Olympus), separated from excitation light by a filter set (ET700SP-2P short-pass filter, 560 DXCR dichroic mirror, ET525/70m-2P emission filter, Chroma Technology) and detected by a GaAsP photomultiplier tube (H11706-40, Hamamatsu Photonics).

### AFM measurements

IT AFM measurements and contact-mode imaging were carried out using an MFP-3D-BIO AFM (Asylum Research/Oxford Instruments) in combination with an inverse optical microscope (IX71S1F-3, Olympus). This guaranteed a precise lateral positioning of the AFM tip. The whole setup was placed on an active vibration isolation table (Halcyonics_i4, Accurion) inside a 1 m^3^ soundproof box to reduce the influence of external noise. All AFM experiments were performed with silicone-nitride cantilevers (MLCT, Cantilever E from Bruker AFM Probes) with a nominal spring constant of 0.1 N/m, a nominal tip radius of 20 nm and a pyramidal tip shape. For each cantilever the spring constant was determined individually using the thermal noise method (Butt and Jaschke, 1995). Native Achilles tendon tissues were snap frozen and cut with a cryotome (Leica CM 1950, Leica) into 20 µm sections. In order to preserve tissue integrity throughout AFM measurements, transparent adhesive tape was used to obtain the tissue sections, which were then attached to a glass slide via a double adhesive tape. Imaging was carried out in air. All images were recorded with a resolution of 512×512 pixels at a line rate of 1 Hz. Image processing and measurements were performed in Gwyddion 2.53. Outliers were removed and a simple plane fit was applied to every image.

Collagen fibers (10-15 µm) in tendons are laying perfectly in the proximodistal axis; therefore, a Fourier transformation of the 90×90 µm^2^ images was performed, showing the angle of the proximodistal axis (pda). The alignment of the collagen fibrils (∼150 nm) was determined by angle measurements with reference to the pda. Then, tissue sections were immersed in Dulbecco's PBS without Mg^2+^/Ca^2+^ (Biochrom AG) during the indentation measurements. Every force map contained 24×25 force-indentation curves equally distributed over an area of 3×3 µm². The vertical tip velocity was constant at 10 µm/s and a setpoint of 3 V was used throughout all measurements. Two different sections per animal were used, and on each slide 3×600 on the central Achilles tendon and 3×600 curves on the lateral part were assessed. No differences were found between the central and the lateral part; therefore, the measurements were merged. In total, 12×600 force curves were assessed for each animal, each genotype and each age group. The Young's modulus was extracted via fitting the Hertz–Sneddon model for a pyramidal shaped tip onto the approach part of the force-indentation curves up to an indentation depth of 500 nm, using a custom software written in MATLAB 2019a (MathWorks). The software automatically corrects the baseline and sets the contact point. Subsequently, stiffness distributions were generated with Igor Pro software (Version 6.3.7.2, WaveMetrics).

### Behavioral tests

Because MLIII gamma equally affects male and female subjects, we performed the behavioral analyses on only male mice to reduce the number of tested animals in accordance with the 3Rs principles for animal welfare. Therefore, ten wild-type and seven *Gnptg^ko^* male mice underwent a longitudinal study to assess different behavioral functions. All tests were performed during the dark cycle of the mice, and the experimental room was illuminated with red light. The strength of the forelimbs was measured in 5- and 7-month-old mice using a GripStrengthMeter system (TSE Systems), as previously described (Morellini and Schachner, 2006). Mice were suspended by the tail and allowed to grasp with the front paws a stainless steel grip attached to a dynamometer. The maximal force applied while pulling the mice until they released the grip was recorded. Mice were tested in three sessions of three trials with an inter-trial interval (ITI) of 10 s and an inter-session interval of 45 min. The mean of three maximal values among the three trials was used for analysis. Locomotor activity and novelty induced behavior were assessed in 4- and 6-month-old mice in the open field test. Briefly, mice were placed in one corner of a white box (50 cm×50 cm and 40 cm high; 20 lux), and their behavior was analyzed for 20 min with EthoVision software (Noldus), as previously described (Brandewiede et al., 2005).

The coordinated motor function of the four limbs was evaluated with the rotarod test (RotaRod, TSE Systems) with mice at the age of 4, 6 and 8 months. Mice were first familiarized with the rotarod by letting them stay for 120 s on the rod rotating at 2 rotations per minute (rpm). Motor learning was assessed by training the mice with an accelerated rotarod protocol of four trials (ITI=45 min), during which the rod accelerated from 4 rpm to 34 rpm within 4.5 min. Possible fatigue- or pain-induced decline in motor performance was tested by leaving the mice on the rod rotating at a constant speed of 32 rpm over three consecutive trials (ITI=45 min). All trials had a maximal duration of 300 s and were interrupted when the mouse fell, or it stopped walking on the rotating rod.

### Patients

Clinical and genetic information on the patients who participated in this study is provided in Tables S1 and S2. Written informed consents on the research procedures were obtained from all patients. The study protocol complies with the Declaration of Helsinki.

To measure joint mobility, a standard mechanical goniometer was placed across the hip, shoulder, elbow, knee, ankle or wrist joint as per standard procedure (Gajdosik and Bohannon, 1987), and maximal angular distances of joint motions (ROMs) were recorded. All the evaluations were performed by the same specialist (an occupational therapist), except for one MLIII alpha/beta patient (evaluation of whom was performed by a physical therapist). Each measurement was performed in triplicate by the same evaluator, and a mean value was calculated.

During surgical hip arthroplasty of Patient 9 (at the age of 43 years) at the Pathology Department, a femoral head specimen was collected and preserved in formalin. Following decalcification in 5% nitric acid for 3 days, the specimen was embedded in paraffin and cut in sagittal orientation to prepare 5 µm thick sections. Staining of the femur head sections was carried out according to standard protocols using Alcian Blue solution (Dinâmica) to detect GAGs and Haematoxylin/Eosin (Merck) to visualize general tissue structure. In addition, Toluidine Blue staining was performed to detect GAGs.

### Statistical analyses

Data are shown as mean±s.d. or mean±s.e.m. Statistical analysis of the data was performed using an unpaired, two-tailed Student’s *t*-test (Microsoft Excel), a two-tailed Mann–Whitney test (GraphPad Prism 8), or a mixed two-way ANOVA with genotype as between factor and time bin (for the open field test) and trial (for the rotarod test) as within groups factors, followed by Sidak's multiple comparisons when appropriate (GraphPad Prism 8). All tests were two-tailed, and differences between the values were considered statistically significant when the *P*-value was ≤0.05.

## Supplementary Material

Supplementary information
