# Supplementary material for: Imbalanced cellular metabolism compromises cartilage homeostasis and joint function in a mouse model of mucolipidosis type III gamma
Source: Dis Model Mech. 2020 Nov 18;13(11):dmm046425. doi: 10.1242/dmm.046425 (PMC7687858; doi:10.1242/dmm.046425)
Supplement: Supplementary information [file dmm-13-046425-s1.pdf]

Supplementary Information

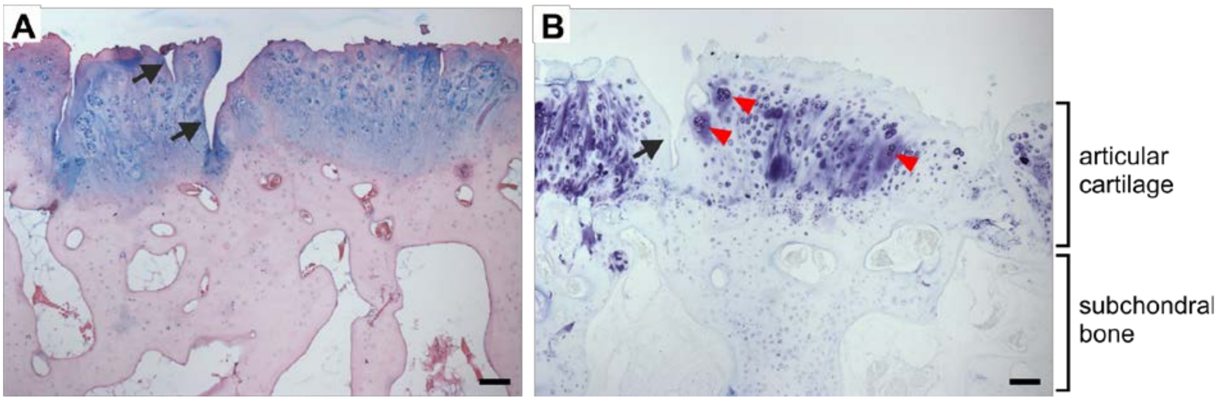

Fig. S1. Histology analysis of an MLIII gamma patient's femur head reveals cartilage degeneration. (A) Alcian blue/Haematoxylin/Eosin and (B) toluidine blue staining of paraffin-embedded sections from a femur head of Patient 9 (at the age of 43 years) diagnosed with MLIII gamma. Sites of cartilage erosion (clefts) are indicated with black arrows. Red arrowheads indicate clusters of chondrocytes. Scale bar: 100 µm.

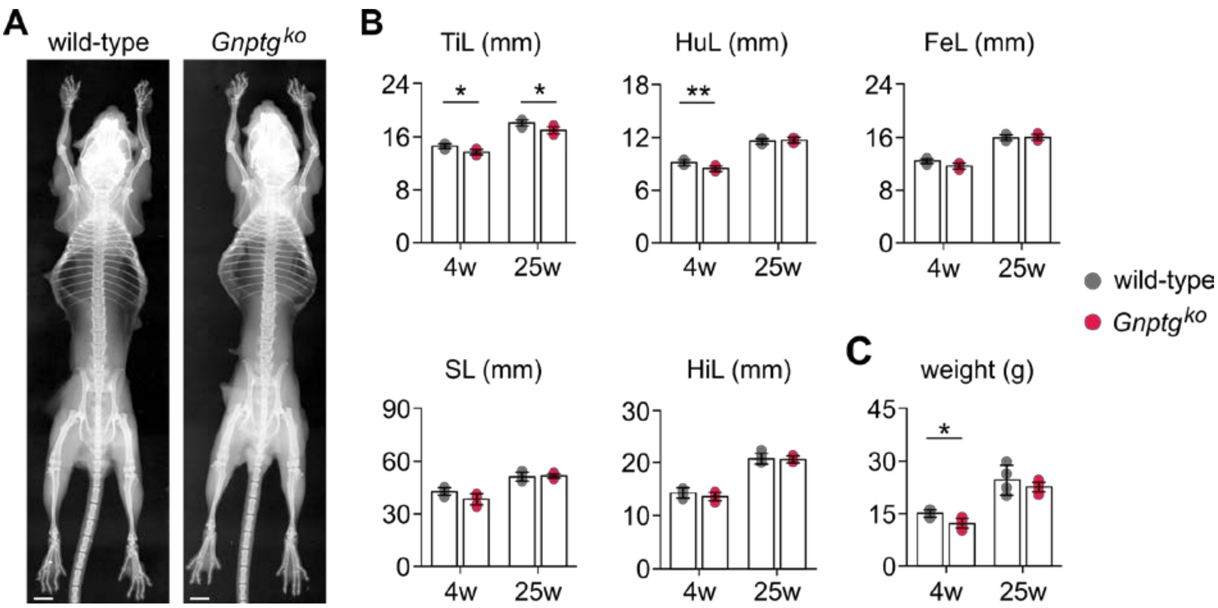

Fig. S2. G*nptg*^{ko} mice display moderate growth retardation. (A) Representative contact radiographs of whole skeletons of 25-weeks-old wild-type and *Gnptg*^{ko} mice. Scale bar: 5 mm. (B) Quantification of tibia length (TiL), humerus length (HuL), femur length (FeL), spine length (SL) and hip length (HiL) of 4- and 25-weeks-old wild-type and *Gnptg*^{ko} mice. (C) Body weight of 4- and 25-weeks-old wild-type and *Gnptg*^{ko} mice. All data are shown as means ± SD ($n \geq 4$). *$P \leq 0.05$, **$P \leq 0.01$ (unpaired two-tailed *t*-test).

Disease Models & Mechanisms • Supplementary information

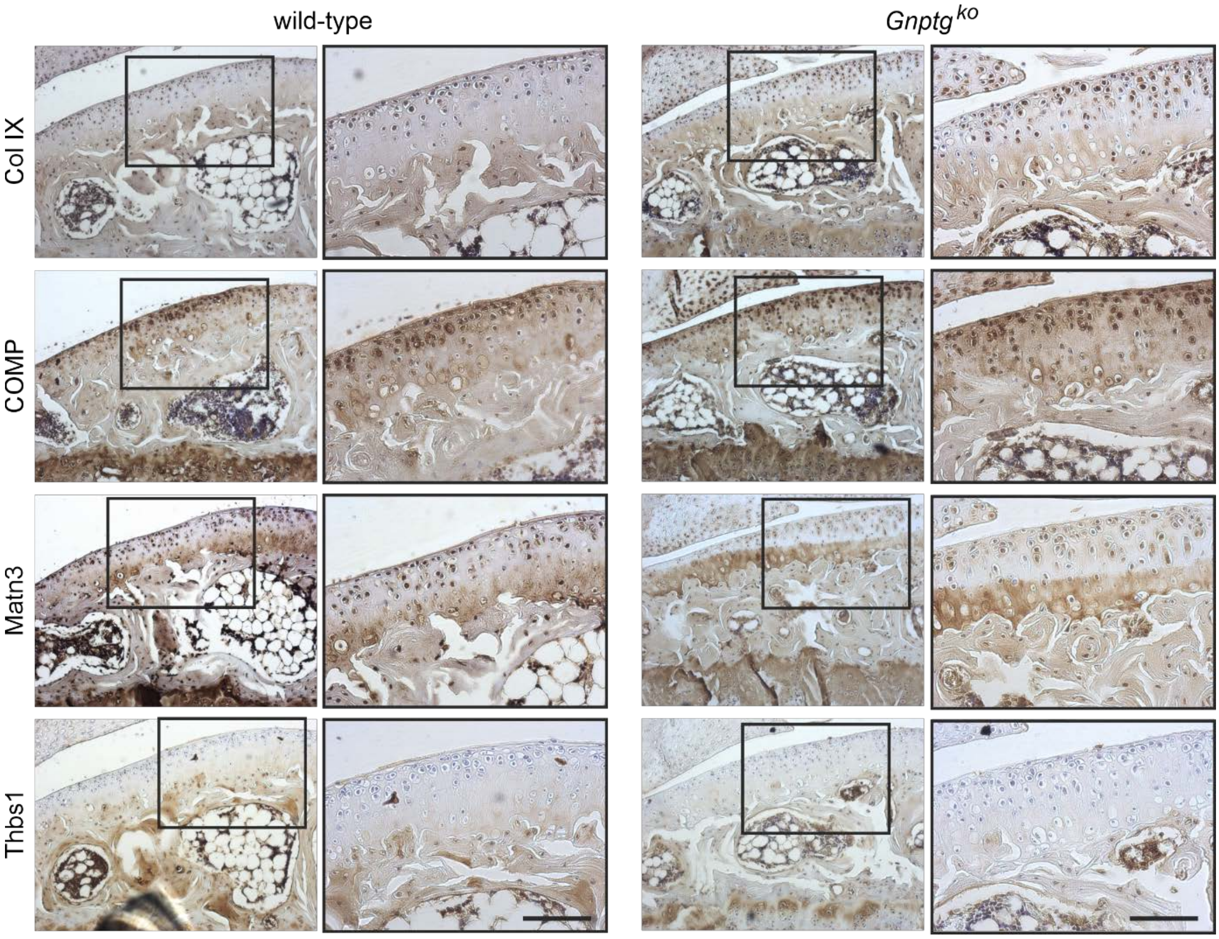

Fig. S3. Abundance of the cartilage ECM proteins is not altered in G*nptg*^{ko} mice. Immunostaining of sagittal paraffin-embedded sections of the knee joint articular cartilage from 60-weeks-old wild-type and *Gnptg*^{ko} mice against collagen type IX (Col IX), cartilage oligomeric matrix protein/thrombospondin-5 (COMP), matrilin-3 (Matn3) and thrombospondin-1 (Thbs1). Representative images of three mice per genotype are shown. Scale bar: 100 µm.

Disease Models & Mechanisms • Supplementary information

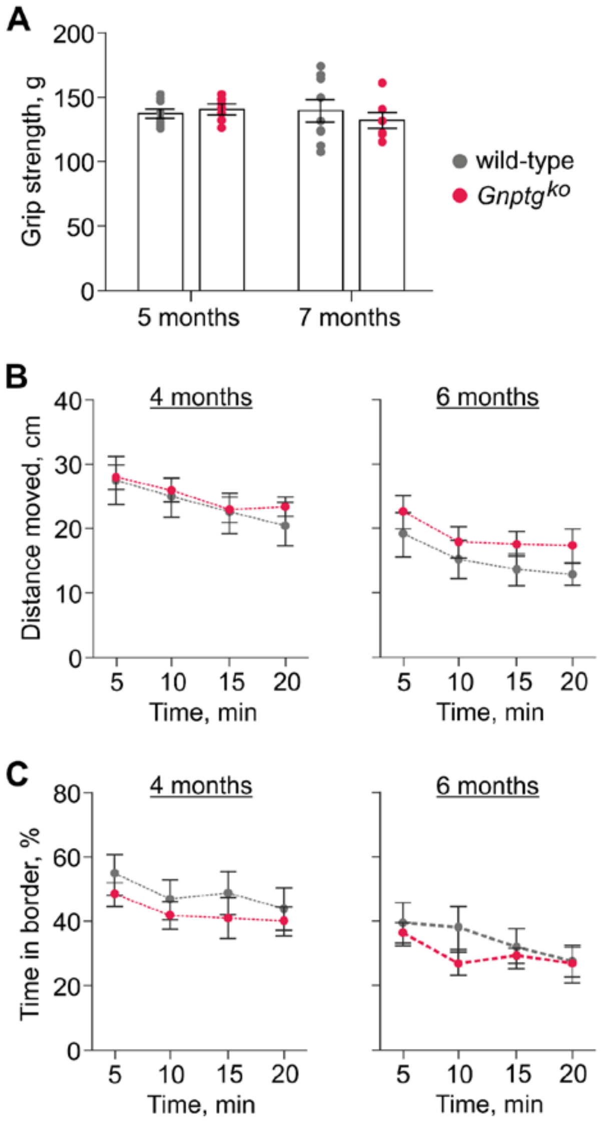

Fig. S4. *Gnptg*^{ko} mice display normal grip strength and behavior in the open field test. (A) Strength of the forelimbs was measured in 5- and 7-months-old *Gnptg*^{ko} and wild-type mice. (B, C) An open field test performed in 4- and 6-months-old *Gnptg*^{ko} and wild-type mice to assess locomotor activity and novelty-induced behavior. Values are shown as means ± SEM (n = 10 for wild-type, n = 7 for *Gnptg*^{ko}).

Disease Models & Mechanisms • Supplementary information

Table S1. Patients with MLII, MLIII alpha/beta and MLIII gamma analyzed in this study

Patient	Age	Sex	Clinical diagnosis	Molecular diagnosis	References
1	33 mo	F	MLII	*GNPTAB*: ?/? *	this study
2	14 mo	M		*GNPTAB*: c.3503_3504del/ c.3503_3504del	[1], [2] (patient 12 in both)
3	61 mo	M		*GNPTAB*: c.3503_3504del/c.2808A>G	[1], [2] (patient 7 in both)
4	48 mo	F		*GNPTAB*: c.3503_3504del/c.1154C>T	[1] (patient 4), [3] (patient 6), [2] (patient 4)
5	19 yrs	M	MLIII alpha/beta	*GNPTAB*: c.3503_3504del/c.1196C>T	[1], [2] (patient 8 in both)
6	14 yrs	M		*GNPTAB*: c.3503_3504del/? *	this study
7	17 yrs	F	MLIII gamma	*GNPTG*: c.244_247dup/c.328G>T	[4] (patient 1), [5] (patient 1), [2] (patient 21)
8	41 yrs	M		*GNPTG*: c.328G>T/ c.328G>T	[6] (patient A), [2] (patient 19)
9	38 yrs	M		*GNPTG*: c.328G>T/c.328G>T	[6] (patient B), [2] (patient 20)

mo, months; yrs, years;
* Patients 1 and 6 were diagnosed clinically and biochemically as MLII and MLIII alpha/beta, respectively.

Table S2. Joint range of motions (°) measurements in patients with MLII, MLIII alpha/beta and MLIII gamma

	Disease	MLII				MLIII alpha/beta		MLIII gamma			Reference values
	Patient	1	2	3	4	5	6	7	8	9	
Shoulder	flexion	117	101	137	137	104	123	160	137	126	150 - 180
	extension	58	71	60	61	68	33	66	67	68	50 - 60
	abduction	93	81	104	105	88	167	110	106	101	180
	internal rotation	31	50	40	70	52	80	50	35	32	70 - 90
	external rotation	81	79	75	88	58	77	70	53	40	90
Elbow	flexion	130	131	128	122	130	133	134	126	121	140 - 150
	extension	20	10	19	25	15	6	0	39	49	0 - 10
Wrist	flexion	70	67	48	39	89	48	60	64	32	60 - 80
	extension	31	55	41	36	66	49	40	20	54	60 - 70
Hip	flexion	127	131	122	114	131	106	125	70	99	100 - 120
	extension	11	19	11	18	14	5	22	16	16	20 - 30
	abduction	37	46	39	39	37	18	22	18	23	40
	adduction	23	23	22	29	27	21	28	22	24	20
	internal rotation	54	71	33	75	58	31	40	23	39	40 - 45
	external rotation	43	37	22	22	32	37	30	15	21	45 - 50
Knee	flexion	136	132	118	131	137	ND	140	130	126	150
	extension	27	21	11	37	10	ND	0	11	10	0 - 10
Ankle	flexion	37	19	30	43	10	11	ND	11	12	40 - 50
	dorsal extension	28	30	10	21	64	41	ND	23	25	20

ND, not determined

Disease Models & Mechanisms • Supplementary information

Table S3 (related to Fig. 2D). Expression of genes encoding lysosomal enzymes in wild-type primary chondrocytes relative to *Gapdh*

Gene	Protein encoded	$\Delta C_{t\ Gene\ -\ Gapdh}$
Ctsb	Cathepsin B	2.62
Ctsc	Cathepsin C	3.01
Ctsl	Cathepsin L	3.03
Ctsd	Cathepsin D	3.23
Ctsz	Cathepsin Z	3.24
Ctsk	Cathepsin K	5.65
Cln5	Ceroid-lipofuscinosis neuronal protein 5	5.66
Tpp1	Tripeptidyl-peptidase 1	6.03
Aga	β-N-acetylglucosaminyl-L-asparaginase	6.08
Gba	Lysosomal acid glucosylceramidase	6.20
Ctss	Cathepsin S	6.56
Neu1	Sialidase-1	6.78
Asah1	Acid ceramidase	6.90
Arsa	Arylsulfatase A	7.06
Plbd2	Putative phospholipase B-like 2	7.24
Gusb	β-Glucuronidase	7.34
Fuca1	α-L-fucosidase	8.21
Arsb	Arylsulfatase B	8.23
Ppt2	Lysosomal thioesterase PPT2	8.56
Ppt1	Palmitoyl-protein thioesterase 1	8.67
Pla2g15	Phospholipase A2 group XV	9.12
Hexb	β-Hexosaminidase	9.33
Glb1	β-Galactosidase	9.41
Lipa	Lysosomal acid lipase	9.52
Man2b1	Lysosomal α-mannosidase	9.67
Acp2	Lysosomal acid phosphatase	9.81
Galns	N-acetylgalactosamine-6-sulfatase	10.01
Prcp	Lysosomal Pro-X carboxypeptidase	10.21
Hyal1	Hyaluronidase-1	10.43
Idua	α-L-Iduronidase	10.55
Dpp7	Dipeptidyl peptidase 2	10.68
Ggh	γ-Glutamyl hydrolase	10.83
Siae	Sialate O-acetylesterase	10.93
Manba	β-Mannosidase	10.99
Acp5	Lysosomal acid phosphatase 5	11.07
Arsk	Arylsulfatase K	11.20

Disease Models & Mechanisms • Supplementary information

Table S4 (related to Fig. 2H). Relative gene expression of chondrocyte markers in wild-type and *Gnptg*^{ko} primary chondrocytes.

**Gene**	**Protein encoded**	**Wild-type**	***Gnptg*^{ko}**
Acan	Aggrecan	1.01 ± 0.03	0.49 ± 0.19
Bgn	Biglycan	1.03 ± 0.05	0.58 ± 0.02
Dcn	Decorin	1.05 ± 0.03	0.62 ± 0.06
Fn1	Fibronectin	1.01 ± 0.04	0.34 ± 0.05
Col2a1	Collagen α1(II) chain	1.02 ± 0.06	0.20 ± 0.03
Col9a1	Collagen α1(IX) chain	1.02 ± 0.09	0.26 ± 0.04
Col10a1	Collagen α1(X) chain	1.01 ± 0.09	0.35 ± 0.01
Gnptg	γ-subunit GlcNAc-1-phosphotransferase	1.02 ± 0.04	0.14 ± 0.01
Gnptab	α/β-subunit GlcNAc-1-phosphotransferase	1.01 ± 0.05	1.01 ± 0.03

Disease Models & Mechanisms • Supplementary information

References

[1] G.K. Cury, U. Matte, O. Artigalás, T. Alegra, R.V. Velho, F. Sperb, M.G. Burin, E.M. Ribeiro, C.M. Lourenço, C.A. Kim, E.R. Valadares, M.F. Galera, A.X. Acosta, I.V.D. Schwartz, Mucolipidosis II and III alpha/beta in Brazil: Analysis of the GNPTAB gene, Gene. 524 (2013) 59–64. https://doi.org/10.1016/J.GENE.2013.03.105.

[2] T. Alegra, F. Sperb-Ludwig, N.R. Guarany, E.M. Ribeiro, C.M. Lourenço, C.A. Kim, E.R. Valadares, M.F. Galera, A.X. Acosta, D.D.G. Horovitz, I.V.D. Schwartz, Clinical Characterization of Mucolipidoses II and III: A Multicenter Study, J Pediatr Genet. 08 (2019) 198–204. https://doi.org/10.1055/s-0039-1697605.

[3] N.F. Ludwig, R.V. Velho, F. Sperb-Ludwig, A.X. Acosta, E.M. Ribeiro, C.A. Kim, D.D. Gandelman Horovitz, R. Boy, M.J. Rodovalho-Doriqui, C.M. Lourenço, E.S. Santos, T. Braulke, S. Pohl, I.V.D. Schwartz, GNPTAB missense mutations cause loss of GlcNAc-1-phosphotransferase activity in mucolipidosis type II through distinct mechanisms, Int. J. Biochem. Cell Biol. 92 (2017) 90–94. https://doi.org/10.1016/j.biocel.2017.09.006.

[4] R.V. Velho, T. Alegra, F. Sperb, N.F. Ludwig, M.L. Saraiva-Pereira, U. Matte, I. V.D. Schwartz, A de novo or germline mutation in a family with Mucolipidosis III gamma: Implications for molecular diagnosis and genetic counseling, Mol. Genet. Metab. Reports. 1 (2014) 98–102. https://doi.org/10.1016/J.YMGMR.2014.01.002.

[5] N.F. Ludwig, F. Sperb-Ludwig, R.V. Velho, I.V.D. Schwartz, Next-generation sequencing corroborates a probable de novo GNPTG variation previously detected by Sanger sequencing, Mol. Genet. Metab. Reports. 11 (2017) 92–93. https://doi.org/10.1016/j.ymgmr.2017.02.002.

[6] R.V. Velho, N.F. Ludwig, T. Alegra, F. Sperb-Ludwig, N.R. Guarany, U. Matte, I. V.D. Schwartz, Enigmatic in vivo GlcNAc-1-phosphotransferase (GNPTG) transcript correction to wild type in two mucolipidosis III gamma siblings homozygous for nonsense mutations, J. Hum. Genet. 61 (2016) 555–560. https://doi.org/10.1038/jhg.2016.13.

Disease Models & Mechanisms • Supplementary information
